# Clinical and Clinical Pathological Presentation of 310 Dogs Affected by Lymphoma with Aberrant Antigen Expression Identified via Flow Cytometry

**DOI:** 10.3390/vetsci9040184

**Published:** 2022-04-13

**Authors:** Elena Celant, Laura Marconato, Damiano Stefanello, Pierangelo Moretti, Luca Aresu, Stefano Comazzi, Valeria Martini

**Affiliations:** 1 Department of Veterinary Medicine and Animal Sciences, University of Milan, Via dell’Università 6, 26900 Lodi, Italy; elena.celant@studenti.unimi.it (E.C.); damiano.stefanello@unimi.it (D.S.); pierangelo.moretti@unimi.it (P.M.); stefano.comazzi@unimi.it (S.C.); 2 Department of Veterinary Medical Sciences, University of Bologna, Via Tolara di Sopra 50, Ozzano dell’Emilia, 40064 Bologna, Italy; laura.marconato@unibo.it; 3 Department of Veterinary Sciences, University of Turin, Largo Braccini 2, Grugliasco, 10095 Turin, Italy; luca.aresu@unito.it

**Keywords:** aberrancy, flow cytometry, immunophenotype, lymphoma

## Abstract

Phenotypic aberrancies have been reported occasionally in canine lymphomas. Here, we retrospectively collected 310 canine lymphomas with an aberrant phenotype detected via flow cytometry and describe their clinical and clinical pathological features at diagnosis. There were 152 T-cell lymphomas not otherwise specified (T-NOS), 101 T-zone lymphomas (TZL), 54 B-cell lymphomas, and 3 cases with two suspected concurrent neoplastic populations. The most represented aberrancies were: CD5-, CD4-CD8-, and CD3- in T-NOS lymphomas, CD21+, CD4-CD8-, and CD3- in TZLs, and CD34+, CD44-, and CD5+ in B-cell lymphomas. Among T-cell lymphomas, the aberrant expression of CD21 was significantly more frequent in TZL and the loss of CD5 and CD44 in T-NOS. More than 75% of dogs were purebred; males outnumbered females; the mean age at diagnosis was 8–10 years, depending on lymphoma subtype. A few dogs were symptomatic at the time of diagnosis, and 30% had peripheral blood abnormalities, in line with what is already reported for the general population of dogs with lymphoma. Further studies are needed to assess the pathogenetic mechanisms underlying each specific antigen aberrancy, as well as the diagnostic and prognostic role.

## 1. Introduction

Immunophenotype represents the cornerstone for disease classification and prediction of prognosis in dogs with lymphoma. In human medicine, abnormal antigen expression and loss of expression of markers that are specific for the cell lineage or maturation stage are defined as “phenotypic aberrancies”, irrespective of their molecular pathogenesis [1]. Phenotypic aberrancies are considered a hallmark of neoplasia and are helpful to discriminate neoplastic from the non-neoplastic resident lymphoid population, to facilitate blood and marrow staging of the disease, as well as the quantification of residual disease after therapy [2]. In dogs, immunocytochemistry and immunohistochemistry are commonly applied to discriminate between T-cell (CD3+ and/or CD5+) and B-cell (CD79a+ and/or CD20+) lymphoma [3,4], but are not suitable to detect phenotypic aberrancies. Conversely, flow cytometry (FC) allows assessing the concomitant expression of different antigens on the same cells, leading to the identification of atypical (aberrant) antigen expression [1,5,6]. The most striking examples of aberrant phenotypes in dogs include T-zone lymphomas (TZL) and extranodal lymphomas [7,8,9]. Indeed, TZL is characterized by the loss of the panleukocyte marker CD45 and the aberrant acquisition of the B-cell marker CD21, both due to the deregulation of gene transcription [10,11], whereas discordant T-cell markers’ expression and loss of both CD4 and CD8 are commonly detected in extranodal T-cell lymphomas [9].

It remains unanswered whether canine lymphomas with aberrant antigen expression represent distinct entities with specific clinical, immunophenotypic, and molecular characteristics or if the aberrancies constitute an incidental phenomenon during malignant transformation. The aim of the present retrospective study is to describe the clinical and clinical pathological features of a large number of canine lymphomas with an aberrant phenotype.

## 2. Materials and Methods

FC data of dogs originally newly diagnosed with nodal or extranodal lymphoma were retrieved from the database of the FC diagnostic service of the Department of Veterinary Medicine and Animal Sciences (University of Milan, Italy), covering the period from January 2009 to December 2019. The diagnosis of lymphoma was based on the clinical suspicion, cytology, and FC of solid lesions, including aspirates from lymph nodes (LN) (*n* = 276), skin (*n* = 14), effusion (*n* = 11), mediastinal masses (*n* = 9), the spleen (*n* = 4), and lesions in the oral cavity (*n* = 3). Aspirates from multiple target lesions were obtained only in a subset of cases, always confirming the same phenotypic aberrancy. Cases were excluded if neoplastic cells tested negative for all lymphoid antigens and if only peripheral blood (PB) and/or bone marrow (BM) samples were analyzed via FC, without any tissue aspirate. Cases were eventually retained in the study if a surface aberrant antigen expression was detected via FC, being defined as loss of expression of panleukocyte markers or lineage-specific markers, co-expression of antigens from different lineages or maturation stages, or co-expression or loss of both CD4 and CD8 by T-cells [8]. Cases were diagnosed as: (1) B-cell lymphoma, if neoplastic cells were CD21+ and stained negative for the majority of T-cell antigens; (2) TZL, if neoplastic cells had a small clear cell appearance by cytological assessment, were small sized, CD45-, and positive for any T-cell marker via FC [8]; and (3) T-cell lymphoma not otherwise specified (T-NOS) if more than one T-cell antigen was expressed and stained positive for CD45. B-cell lymphomas were considered aberrant if neoplastic cells were CD45- or CD44- (panleukocytes markers), CD34+ (precursor cells marker), or CD5+, CD3+, CD4+, or CD8+ (T-cell markers). T-cell lymphomas were considered aberrant if neoplastic cells were CD45- or CD44-, CD34+, CD21+ (B-cell marker), or CD5- or CD3-, CD4+CD8+, or CD4-CD8-. A subset of CD45- T-cell lymphomas had medium-to-large-sized cells and a high number of mitoses at cytological evaluation: those cases were considered inconsistent with TZL and finally included in the T-NOS group. Fluorescence intensity data were not considered in the present study.

All samples were processed for FC according to published protocols [5], with a multi-color approach, using antibodies from the list shown in Table 1. However, both the antibody panel and the combination of fluorochromes applied to the samples varied over time, depending on their availability and on the operator’s preferences. CD4 and CD8 were always tested in a single tube to assess their co-expression. For the other antigens, the co-expression on the surface of neoplastic cells was assessed by concomitant use of different antibodies in the same tube (conjugated with different fluorochromes) or with multi-step analysis among different tubes (for example, assessment of different antigens’ expression on large-sized cells or on CD45- cells in different tubes). Samples were acquired with a BD FACScalibur (Becton Dickinson, San Josè, CA, USA) or a Mindray Bricyte E6 (Mindray, Shenzhen, China) flow cytometer and analyzed with the specific software CellQuest (Becton Dickinson, San Josè, CA, USA) or MRFlow (Mindray, Shenzhen, China), respectively.

For each included case, the following data were retrieved in the database or by contacting the referring veterinarian: breed (pure, mixed), sex (male, female), age at diagnosis (years), presence of symptoms (yes, no), presence of any concomitant disease (yes, no; if present, final diagnosis), enlarged LNs (none, peripheral, intracavitary alone), histopathological diagnosis (yes, no; if present, which one), cytologic spleen involvement (yes, no), cytologic liver involvement (yes, no), extranodal involvement (yes, no; if present, lesion site), FC PB infiltration (%), FC BM infiltration (%), and hematologic abnormalities (anemia, thrombocytopenia, leukocytosis, leukopenia; yes, no). The degree of PB and BM infiltration was defined as the percentage of nucleated cells showing the same FC morphological and phenotypic properties of the neoplastic cells found in the solid lesion [8]. No criteria were provided to the referring veterinarians to define symptomatic dogs; however, the presence of enlarged LNs, spleen, or liver (alone or in combination) was not considered sufficient to classify dogs as symptomatic (Substage B).

All data were included in an electronic datasheet, and descriptive statistics were calculated. Contingency tables were prepared, and a Pearson chi-squared test was used to assess whether aberrancies had a different prevalence between TZL and T-NOS lymphomas. All analyses were performed with a specific statistical software (SPSS v27.0 for Windows, IBM Corp., Endicott, NY, USA). Significance was set at *p* ≤ 0.05 for the Pearson chi-squared test.

## 3. Results

Among 2612 dogs initially included, 310 fulfilled the inclusion criteria. Among them, 49.0% (*n* = 152) were finally categorized as T-NOS lymphomas, 32.6% (*n* = 101) as TZL, 17.4% (*n* = 54) as B-cell lymphomas, whereas 1.0% (*n* = 3) cases had two different suspected neoplastic populations.

Overall, loss of CD45 occurred in 37.1% of the tested cases (*n* = 114/307), loss of CD5 expression in T-cells in 35.8% (*n* = 87/243), loss of both CD4 and CD8 in T-cells in 33.2% (*n* = 80/241), aberrant expression of CD21 in T-cells in 27.9% (*n* = 69/247), expression of CD5 in B-cells in 23.1% (*n* = 12/52), loss of CD3 in T-cells in 20.5% (*n* = 47/229), expression of CD34 in 12.9% (*n* = 35/272), loss of CD44 in 12.0% (*n* = 19/158), co-expression of both CD4 and CD8 in T-cells in 8.3% (*n* = 20/241), expression of CD3 in B-cells in 3.0% (*n* = 1/33), and expression of CD4 and CD8 in B-cells in 2.6% (*n* = 1/38) each.

The prevalence of single aberrancies according to lymphoma subtype is shown in Table 2. The aberrant CD21 expression was significantly more frequent in TZL than in T-NOS lymphomas (*p* < 0.001), whereas the loss of CD5 and CD44 was more frequent in the latter (*p* < 0.001 and *p* = 0.004, respectively).

### 3.1. T-NOS Lymphomas

Among dogs with T-NOS lymphoma, 116 (80.6%) were purebred and 28 (19.4%) were mixed-breed. Seventy-eight (53.8%) were males and sixty-seven (46.2%) were females. The mean age was 7.9 ± 3.0 years (median 8; range, 1–16). Purebred dogs included: Boxer (*n* = 21, 18.1%), Labrador Retriever (*n* = 10, 8.6%), Golden Retriever (*n* = 7, 6.0%), Beagle and German Shepherd (*n* = 5, 4.3% each), Australian Shepherd and English Bulldog (*n* = 4, 3.4% each), and Cane Corso, Dogue de Bordeaux, Dachshund, English Setter, French Bulldog, Pitbull, Rottweiler, and Yorkshire terrier (*n* = 3, 2.6% each), and 28 breeds were represented only twice (1.7%) or once (0.9%) Thirty-eight (34.9%) dogs were symptomatic at diagnosis, and ten (20%) had concomitant diseases. Ninety-two (80.7%) dogs had peripheral lymphadenomegaly; eighteen (15.8%) had intracavitary lymphadenomegaly alone; four (3.4%) dogs had no lymphadenomegaly, and the primary lesion was located in the skin (*n* = 2), stomach and prostate (*n* = 1), or lung and retroperitoneum (*n* = 1).

Histopathology was available for 15 cases (*n* = 9 peripheral T-cell lymphoma (PTCL), *n* = 2 mycosis fungoides, *n* = 2 small lymphocytic lymphoma, *n* = 1 non-epitheliotropic cutaneous lymphoma, and *n* = 1 lymphoblastic lymphoma). The spleen was infiltrated in 15 (35.7%) cases and the liver in 14 (34.1%). Extranodal involvement was found in 54 (74%) dogs (*n* = 13 skin, *n* = 13 mediastinum, *n* = 13 pleural and/or peritoneal effusion, *n* = 6 oral cavity, *n* = 3 bowel, and *n* = 1 lung, and *n* = 5 dogs had multiple extranodal sites).

Regarding bloodwork, 35 (29.9%) dogs were anemic, 47 (41.2%) were thrombocytopenic, 33 (28.2%) had leukocytosis, and 6 (5.1%) had leukopenia. The mean PB infiltration obtained on 110 dogs was 8.3 ± 13.5% (median, 2.8%; range, 0.1–68.0%). The mean BM infiltration obtained on 71 dogs was 8.3 ± 16.7% (median, 1.1%; range, 0.1–80.1%).

**Figure 1:**
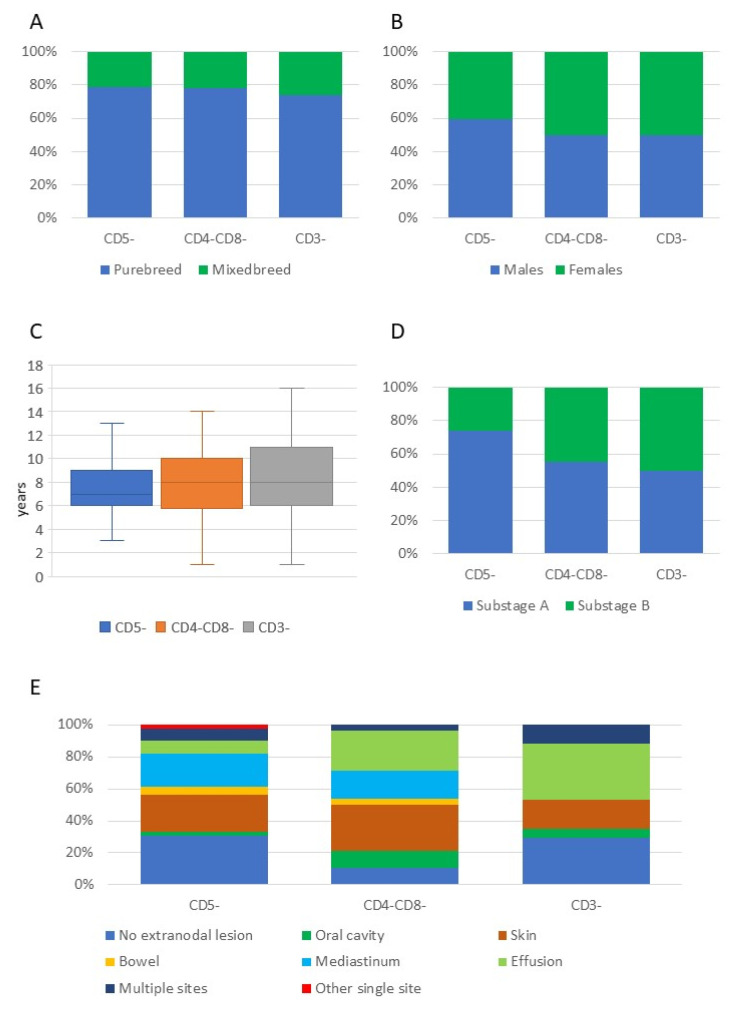
Breed (**A**), sex (**B**), age (**C**), substage (**D**), and presence of extranodal lesions (**E**) in 152 dogs with T-cell lymphoma not otherwise specified, according to the three most common phenotypic aberrancies.

Breed, sex, age, clinical substage, and presence of extranodal lesions in dogs with the three most common phenotypical aberrancies are shown in Figure 1. All clinical and clinical pathological data of dogs with T-NOS lymphoma, according to the phenotypic aberrancies, are shown in Supplementary Figure S1 and Tables S1 and S4.

### 3.2. TZL

Among dogs with TZL, 74 (75.5%) were purebred and 24 (24.5%) were mixed-breed. Fifty (51%) were males and forty-eight (49%) were females. The mean age was 9.6 ± 2.5 years (median, 10; range, 4–15). Purebred dogs included: Jack Russell (*n* = 9, 12.2%), Cane Corso (*n* = 8, 10.8%), Boxer (*n* = 6, 8.1%), Cavalier King Charles Spaniel (*n* = 5, 6.8%), French Bulldog and Labrador Retriever (*n* = 4, 5.4% each), and Bullmastiff and Shi-Tzu (*n* = 3, 4.1% each), and 28 breeds were represented twice (2.7%) or once (1.4%).

Thirteen (19.7%) dogs were symptomatic at diagnosis, and twelve (38.7%) had concomitant diseases. Seventy-two (98.6%) had peripheral lymphadenomegaly, and one (1.4%) had intracavitary lymphadenomegaly. No dog lacking lymphadenomegaly was reported.

Histopathology was obtained in 17 cases (*n* = 15 TZL, *n* = 1 epitheliotropic lymphoma; in 1 case, a T-cell lymphoma was diagnosed, but the WHO subtype was not reported). The spleen was infiltrated in 13 (54.2%) cases and the liver in 11 (45.8%). Extranodal involvement was found in 17 (48.6%) dogs (*n* = 7 skin, *n* = 6 oral cavity, *n* = 1 bowel, *n* = 1 pancreas, and 2 dogs had multiple extranodal sites).

When considering bloodwork results, 20 (25.6%) dogs were anemic, 10 (12.7%) were thrombocytopenic, 27 (34.2%) had leukocytosis, and 2 (2.5%) had leukopenia. The mean PB infiltration obtained on 74 dogs was 27.7 ± 22.2% (median, 21.3%; min–max 0.1–88.4%). The mean BM infiltration obtained on 43 dogs was 7.6 ± 10.0% (median, 2.7%; min–max, 0.1–46.4%).

**Figure 2:**
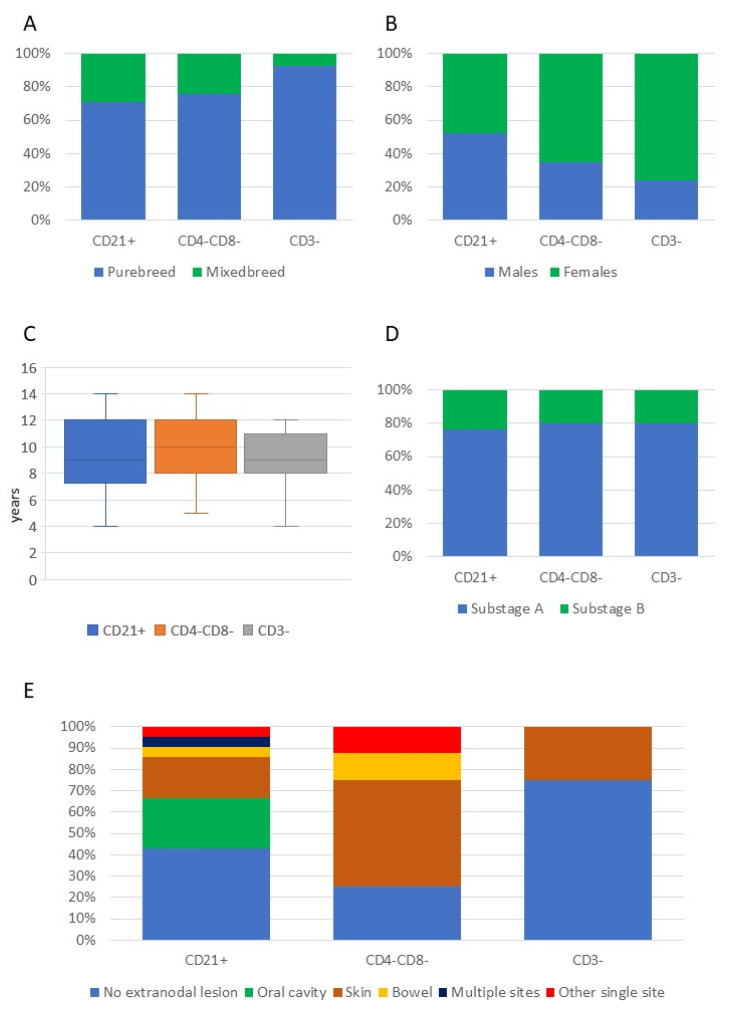
Breed (**A**), sex (**B**), age (**C**), substage (**D**), and presence of extranodal lesions (**E**) in 101 dogs with T-zone lymphoma, according to the three most common phenotypic aberrancies.

Breed, sex, age, clinical substage, and presence of extranodal lesions in dogs with the three most common phenotypical aberrancies are shown in Figure 2. All clinical and clinical pathological data of dogs with TZL, according to the phenotypic aberrancies, are shown in Supplementary Figure S2 and Tables S2 and S4.

### 3.3. B-Cell Lymphomas

Among dogs with B-cell lymphoma, 41 (82%) were purebred and 9 (18%) were mixed-breed. Twenty-nine (56.9%) were males and twenty-two (43.1%) were females. The mean age was 8.7 ± 3.2 years (median, 8 years; min–max 3–15 years). Purebred dogs included: German Shepherd (*n* = 4, 9.8%) and Border Collie, Cocker Spaniel, Doberman Pinscher, and Labrador Retriever (*n* = 3, 7.3% each), and 21 breeds were represented twice (4.9%) or once (2.4%).

Six (18.2%) dogs were symptomatic at diagnosis, and seven (36.8%) had concomitant diseases. All dogs (100%) had peripheral lymphadenomegaly. Histopathology was obtained in six cases (*n* = 3 diffuse large B-cell lymphoma (DLBCL) and *n* = 3 marginal-zone lymphoma (MZL)). The spleen was infiltrated in 15 (88.2%) dogs and the liver in 10 (66.7%). Extranodal infiltration was found in 4 (26.7%) dogs (one each of skin, mediastinal mass, bowel, and peritoneal effusion).

In this group of dogs, 12 (28.6%) were anemic, 11 (26.8%) were thrombocytopenic, and 12 (29.3%) had leukocytosis. The mean PB infiltration obtained on 38 dogs was 6.2 ± 12.0% (median, 1.5%; min–max 0.1–49.8%). The mean BM infiltration obtained on 25 dogs was 5.5 ± 6.4% (median, 3.4%; min–max 0.1–22.1%).

**Figure 3:**
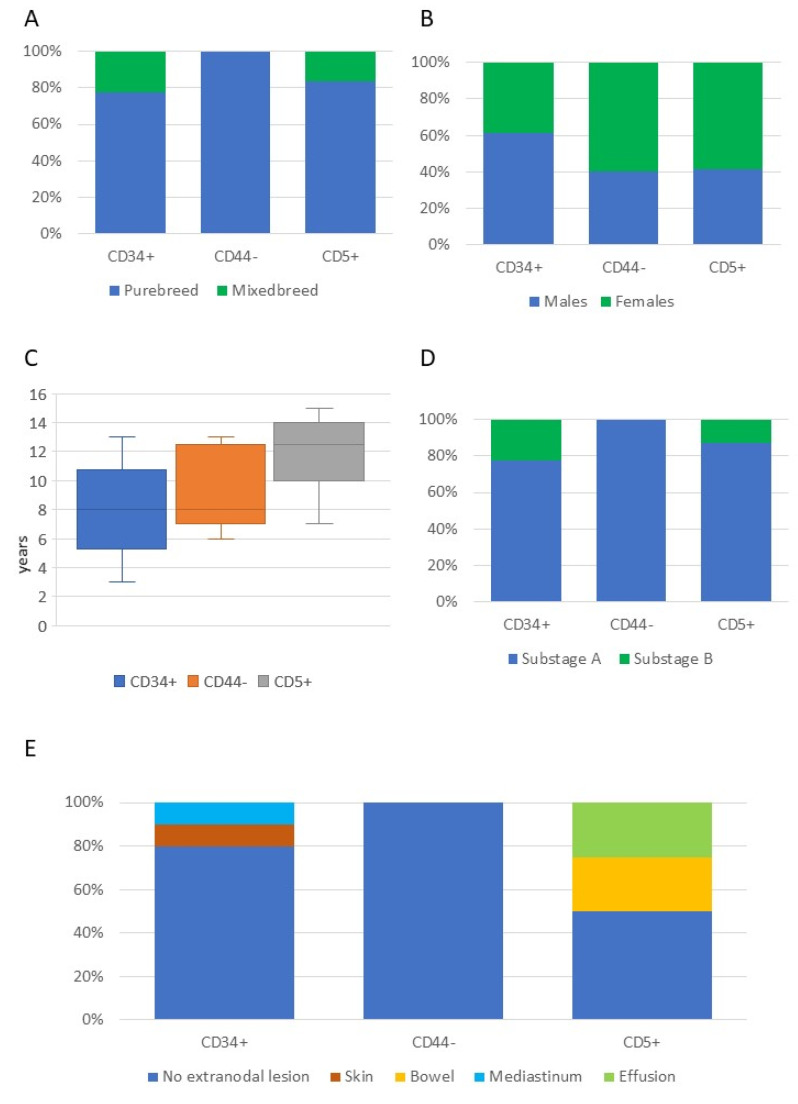
Breed (**A**), sex (**B**), age (**C**), substage (**D**), and presence of extranodal lesions (**E**) in 54 dogs with B-cell lymphoma, according to the three most common phenotypic aberrancies.

Breed, sex, age, clinical substage, and presence of extranodal lesions in dogs with the three most common phenotypical aberrancies are shown in Figure 3. All clinical and clinical pathological data of dogs with B-cell lymphoma, according to the phenotypic aberrancies, are shown in Supplementary Figure S3 and Tables S3 and S4.

### 3.4. Double Suspected Neoplastic Populations

Three dogs were diagnosed with a double neoplastic population.

The first dog was a 15-year-old mixed breed female, asymptomatic at diagnosis and with a single enlarged submandibular LN. The dog had a concomitant extramedullary plasmacytoma. FC on an LN aspirate revealed a prevalent population of medium-sized CD45+CD8+ T-cells and a less represented population of small-sized CD45- T-cells (TZL). Histopathology confirmed the presence of a peripheral T-cell lymphoma (PTCL). PB was infiltrated by CD45- T-cells (4.8%).

The second dog was an 11-year-old male Shi-Tzu with an enlarged submandibular LN. FC on an LN aspirate revealed a prevalent population of CD45+CD8+ T-cells and a less represented population of small-sized CD45- T-cells (TZL). The dog had thrombocytopenia and leukocytosis and a 54% PB infiltration by CD45- T-cells.

The third dog was a 13-year-old female Beagle with peripheral lymphadenomegaly. FC on an LN aspirate revealed a prevalent population of large-sized B-cells (large B-cell lymphoma) and a less represented population of CD45-CD21+ T-cells (TZL). PB was infiltrated by CD45- T-cells (2.1%), but not by large B-cells. Based on the above, this dog was diagnosed with concomitant large B-cell lymphoma and TZL.

## 4. Discussion

Phenotypic aberrancies have occasionally been reported in canine lymphoma, mainly in TZL and extranodal lymphomas [7,8,9]. To the authors’ knowledge, this is the first study specifically focusing on clinical and clinical pathological features of a large population of dogs with lymphoma showing an aberrant phenotype.

T-cell lymphomas, including TZL and T-NOS, were the most frequent. The higher frequency of phenotypic aberrancies among T-cell lymphomas has already been documented [5,6]. The reason behind this finding may be attributable to the reduced number of canine-specific or cross-reactive antibodies commercially available that target B-cell antigens, thereby underestimating B-cell lymphoma aberrancies. Additionally, a higher tendency to lineage infidelity in T-cell neoplasms should also be considered, as occurs in human medicine, where aberrancies are more frequently reported in PTCL [12,13] than in DLBCL [14,15].

More than 75% of dogs were purebred; males overnumbered females; mean age at diagnosis was 8–10 years, depending on the lymphoma subtype. Only a minority of dogs was symptomatic at the time of diagnosis, and 30% had peripheral blood abnormalities (including anemia, thrombocytopenia, or leukocytosis). Most of these findings are in line with previous data [16,17,18,19,20,21].

Previous results showed that 75% of dogs with indolent T-cell lymphoma and 88% of dogs with aggressive T-cell lymphoma were symptomatic at diagnosis [18]. Mizutani and colleagues reported <20% symptomatic subjects in a group of dogs with TZL [22]. In the current study, only 20% of dogs with TZL and 35% of dogs with T-NOS lymphoma were symptomatic at diagnosis. A combination of different factors may explain these discrepancies, including different inclusion criteria, study design, and population size. Thus, the presence of aberrant antigen expression by lymphoma cells cannot be anticipated by signalment, the presence of symptoms, or hematological abnormalities.

When considering TZL and T-NOS lymphomas, a significantly different prevalence of specific phenotypic aberrancies was found: aberrant CD21 expression was more common in TZL, whereas loss of CD5 and CD44 were more common in T-NOS. The loss of CD45 expression, one of the diagnostic criteria for TZL applied in the present study, was found only in 5% of T-NOS lymphomas. The loss of CD45, as well as the aberrant expression of CD21 are well known in TZL [7,8], although both CD45+ TZL and CD45- T-NOS lymphomas have occasionally been reported [23,24]. Loss of CD5 expression has been reported in 43% of CD45+CD4+ T-cell lymphomas [25], whereas data concerning the expression of CD44 in different T-cell lymphoma subtypes are not available. Based on the above, it may be speculated that the combined examination of these four antigens may further improve the diagnostic role of FC in discriminating between TZL and other T-cell subtypes. Unfortunately, the histopathologic diagnosis was not available for the vast majority of cases, thus preventing us from testing this hypothesis.

Contrary to the four aberrancies described above, the CD4-CD8- phenotype had a similar distribution in dogs with T-NOS lymphoma and TZL, representing 34% and 31% of cases, respectively. According to the published literature, 20% of dogs with CD4-CD8- T-cell lymphoma had cutaneous involvement [21], and the CD4-CD8- T-cell phenotype was represented in 20–40% of dogs with cutaneous lymphomatous lesions [9,26]. In our caseload, the CD4-CD8- phenotype was found in 60% of dogs with cutaneous involvement and in <20% of dogs without extranodal involvement in both the T-NOS and TZL cases. Thus, the occurrence of the CD4-CD8- phenotype seems to be common in canine cutaneous lymphoma and vice versa, but a possible role in the discrimination among different T-cell subtypes is questionable. It might be also true that the CD4-CD8- phenotype does not represent a true phenotypic aberrancy in canine cutaneous lymphoma, but rather a neoplastic proliferation of a resident population characterized by this peculiar phenotype. The phenotypic analysis of non-neoplastic lymphocytes residing in the skin is warranted to solve this issue.

Concerning B-cell lymphomas, CD34 expression, loss of CD44, and expression of CD5 were the most represented aberrancies in our caseload. CD34 expression has already been documented in canine B-cell lymphomas [5,6] and its prognostic value investigated and excluded [27]. Conversely, loss of CD44 expression has never been reported. In human DLBCL, its negative stain is a positive prognostic factor [28,29]. In dogs, a negative prognostic influence of high CD44 expression has been reported, although only the transcript amount rather than the protein expression was tested [30]. Looking at the clinical data of dogs presenting with CD5+ B-cell lymphoma, the age at diagnosis appears higher than in dogs without this phenotypic aberrancy (median 12.5 and 8 years, respectively; Figure 3 and Supplementary Table S4). In human DLBCL, the expression of CD5 is associated with older age at diagnosis [31], representing a negative prognostic factor [32,33]. Further studies are needed to assess the prognostic value of CD5 expression and loss of CD44 in old dogs with B-cell lymphoma.

In three cases, a double suspected neoplastic cell population was found via FC. In two dogs, both suspected neoplastic populations had a T-cell phenotype and only differed for CD45 expression. This is likely due to the concomitant presence of two neoplastic clones within the same disease. In the third case, the concomitant presence of B-cell lymphoma and TZL was documented via FC. Two similar clinical cases have been recently described [34,35]. Interestingly, only CD45- cells were found in the PB of all three dogs, suggesting their tendency to invade vessels, as already hypothesized based on the high frequency of PB infiltration in TZL cases [36].

The retrospective nature of this study represents the main pitfall. Indeed, the antibody panel varied among samples, reducing the number of cases tested for each specific aberrancy, and in most cases, the information provided by the referring veterinarians was fragmentary. Furthermore, different staging procedures were adopted by the numerous clinicians involved. Finally, the lack of histopathologic data and the incomplete staging procedures adopted in most cases prevented us from assessing possible associations between specific aberrancies and lymphoma histopathologic subtype or stage at diagnosis.

Most of the antigens tested in the present caseload cannot be analyzed via immunohistochemistry (IHC) due to lack of antibodies available for formalin-fixed paraffin-embedded samples. Consequently, a high number of phenotypic aberrancies would be lost performing only IHC. Still, histopathology plus IHC remains the gold standard technique to classify canine lymphoma according to the WHO system [37]. Thus, we support the concomitant use of FC and IHC for more detailed characterization of canine lymphoma cases.

## 5. Conclusions

In conclusion, this is the first report of a large number of dogs with lymphoma showing aberrant antigen expression. Clinical and clinical pathological presentation at diagnosis were in line with what is generally reported for canine lymphoma. The potential role of the combined assessment of CD45, CD21, CD5, and CD44 in predicting T-cell subtypes should be further addressed, and future studies are needed to assess both the biological mechanisms underlying atypical phenotypes and the clinical relevance.

## Figures and Tables

**Table 1:** Antibodies used for flow cytometric immunophenotyping of suspected canine lymphoma samples.

Target	Antibody Clone	Conjugations	Source	Specificity
CD45	YKIX716.13	FITC ALEXAFLUOR488 ALEXAFLUOR647	Biorad, Oxford, UK	All leukocytes
CD44	IM7	FITC	BDPharmingen, San Diego, CA, USA	All hematopoietic cells
CD3	CA17.2A12	FITC	Biorad	T-cells
CD5	YKIX322.3	FITC PE	Biorad	T-cells
CD4	YKIX302.9	FITC ALEXAFLUOR647	Biorad	T-helper cells and neutrophils
CD8	YCATE55.9	PE	Biorad	T-cytotoxic cells
CD21	CA2.1D6	PE ALEXAFLUOR647	Biorad	Mature B-cells
CD34	1H6	PE	BD Pharmingen	Precursors

**Table 2:** Prevalence of specific antigen aberrancies among 310 dogs with lymphoma, according to the lymphoma subtype.

Aberrancy	Aberrant/Tested Cases (%)		
	T-NOS Lymphoma	T-Zone Lymphoma	B-Cell Lymphoma
Loss of CD45	8/152 (5.3)	101/101 (100.0)	5/54 (9.3)
Loss of CD44	12/66 (18.2)	2/72 (2.8)	5/20 (25.0)
Expression of CD34	1/124 (0.8)	1/96 (1.0)	33/52 (63.5)
Loss of CD5	87/144 (60.4)	0/99 (0.0)	
Expression of CD5			12/52 (23.1)
Loss of CD3	33/145 (22.8)	14/84 (16.7)	
Expression of CD3			1/33 (3.0)
CD4/CD8 double-positive	16/148 (10.8)	4/93 (4.3)	
CD4/CD8 double-negative	51/148 (34.5)	29/93 (31.2)	
Expression of CD4			1/38 (2.6)
Expression of CD8			1/38 (2.6)
Expression of CD21	1/150 (0.7)	68/97 (70.1)	

T-NOS = T-cell lymphoma not otherwise specified.

## Data Availability

The data presented in this study are available upon reasonable request from the corresponding author.

## Supplementary Materials

The following supporting information can be downloaded at: https://www.mdpi.com/article/10.3390/vetsci9040184/s1, Figure S1: Clinical and clinical pathological features of 152 dogs with T-cell lymphoma not otherwise specified, according to specific phenotypic aberrancies; Figure S2: Clinical and clinical pathological features of 101 dogs with T-zone lymphoma, according to specific phenotypic aberrancies; Figure S3: Clinical and clinical pathological features of 54 dogs with B-cell lymphoma, according to specific phenotypic aberrancies; Table S1: Clinical and clinical pathological features of 152 dogs with T-cell lymphoma not otherwise specified with aberrant antigen expression; Table S2: Clinical and clinical pathological features of 101 dogs with T-zone lymphoma with aberrant antigen expression.; Table S3: Number and frequencies of clinical and clinical pathological features of 54 dogs with B-cell lymphoma with aberrant antigen expression; Table S4: Age and peripheral blood and bone marrow infiltration in 310 dogs with lymphoma with aberrant antigen expression, according to lymphoma subtype.
