# Supplementary material for: Clinical and Clinical Pathological Presentation of 310 Dogs Affected by Lymphoma with Aberrant Antigen Expression Identified via Flow Cytometry
Source: Vet Sci. 2022 Apr 13;9(4):184. doi: 10.3390/vetsci9040184 (PMC9032799; doi:10.3390/vetsci9040184)
Supplement: Supplementary file 1 [file vetsci-09-00184-s001.zip › Supplementary Figure S1.pdf]

Supplementary Figure S1

Clinical and clinical-pathological data of 152 dogs diagnosed with T-cell lymphoma not otherwise specified (T-NOS), according to specific phenotypic aberrancies

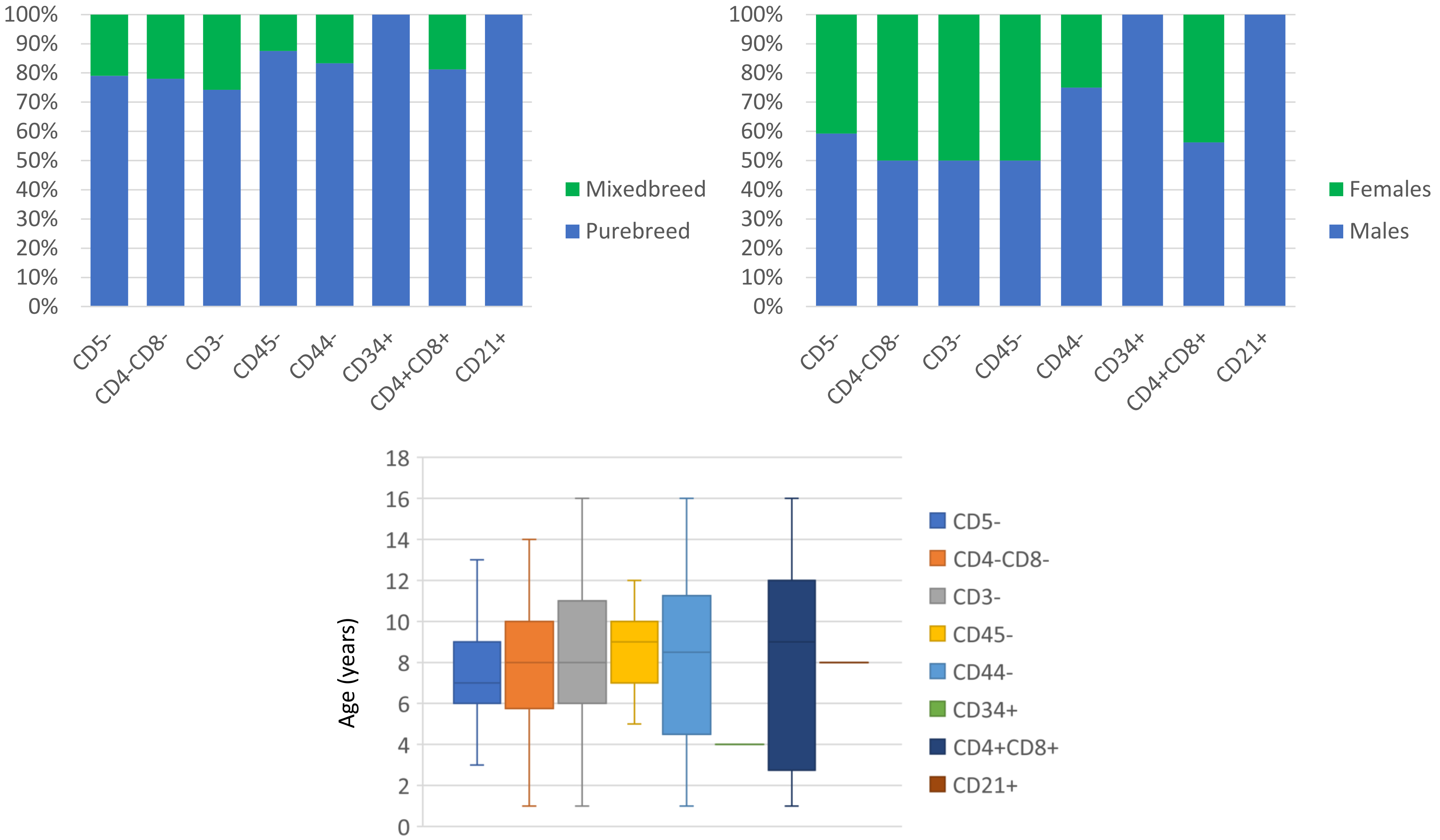

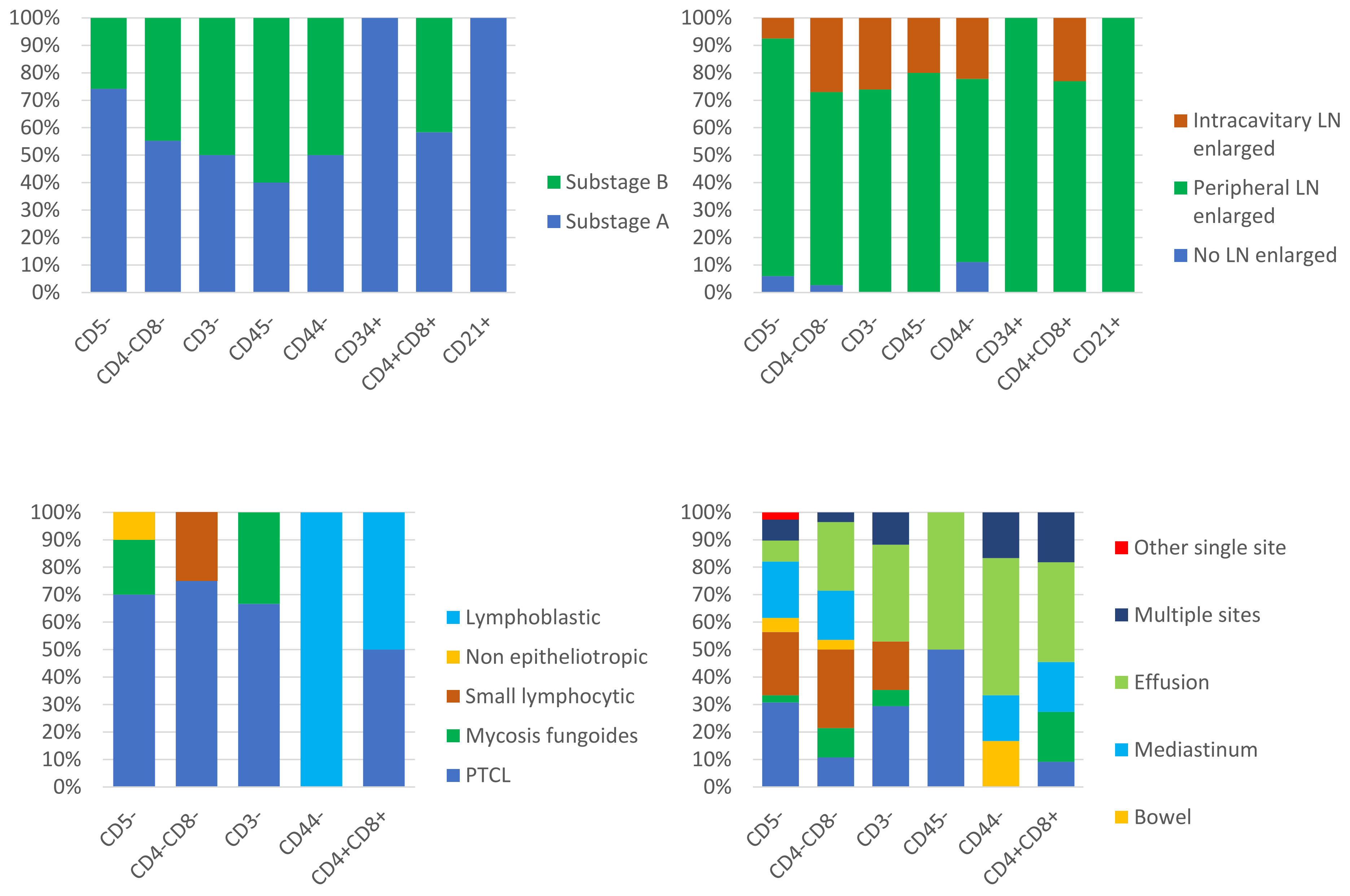

100%
90%
80%
70%
60%
50%
40%
30%
20%
10%
0%
CD5-
CD4-CD8-
CD3-
CD45-
CD44-
CD34+
CD4+CD8+
CD21+
Substage B
Substage A
Intracavitary LN enlarged
Peripheral LN enlarged
No LN enlarged
Lymphoblastic
Non epitheliotropic
Small lymphocytic
Mycosis fungoides
PTCL
Other single site
Multiple sites
Effusion
Mediastinum
Bowel

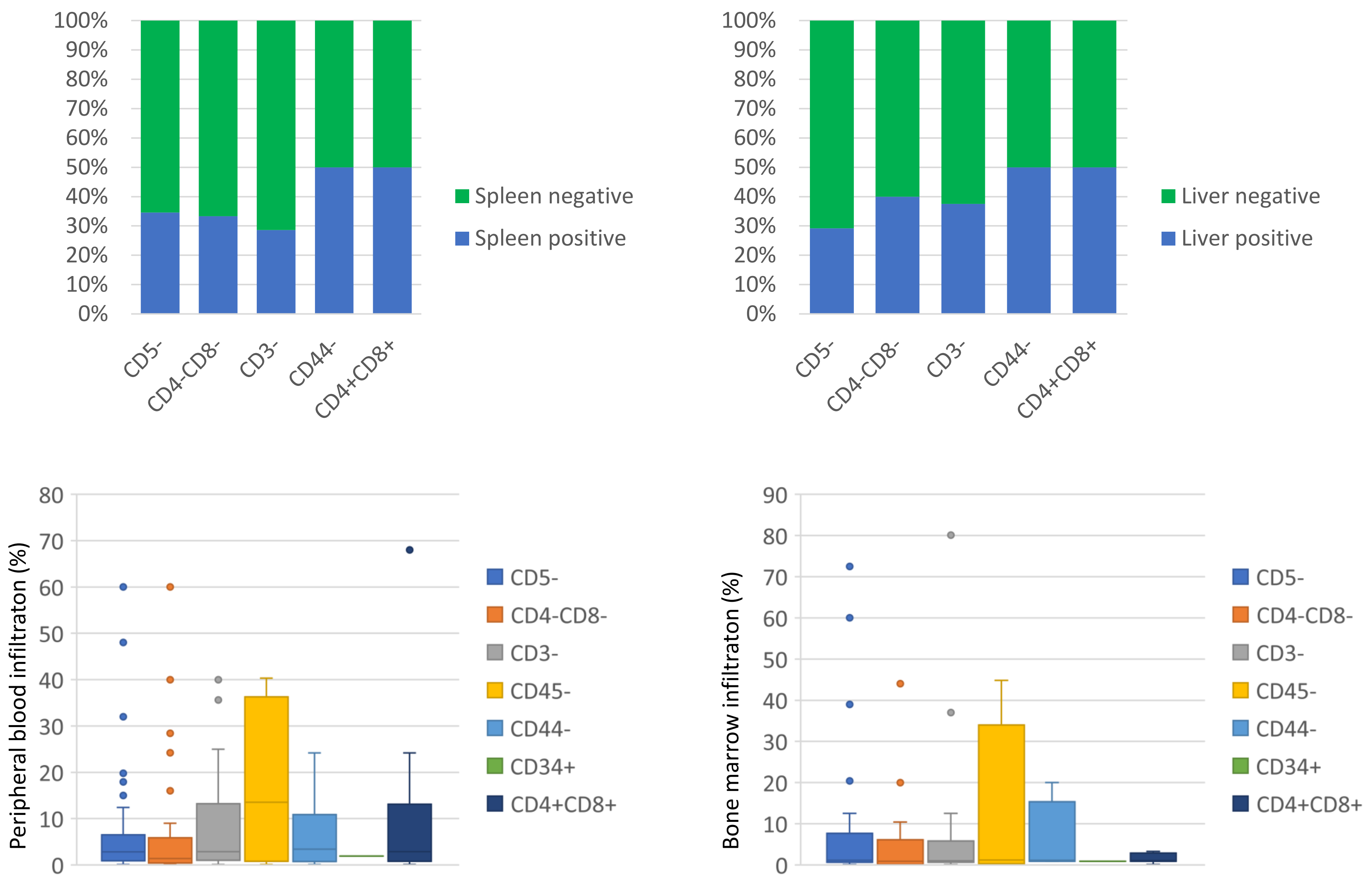
100%
90%
80%
70%
60%
50%
40%
30%
20%
10%
0%
CD5-
CD4-CD8-
CD3-
CD44-
CD4+CD8+
Spleen negative
Spleen positive
Liver negative
Liver positive
Peripheral blood infiltraton (%)
80
70
60
50
40
30
20
10
0
CD5-
CD4-CD8-
CD3-
CD45-
CD44-
CD34+
CD4+CD8+
Bone marrow infiltraton (%)
90

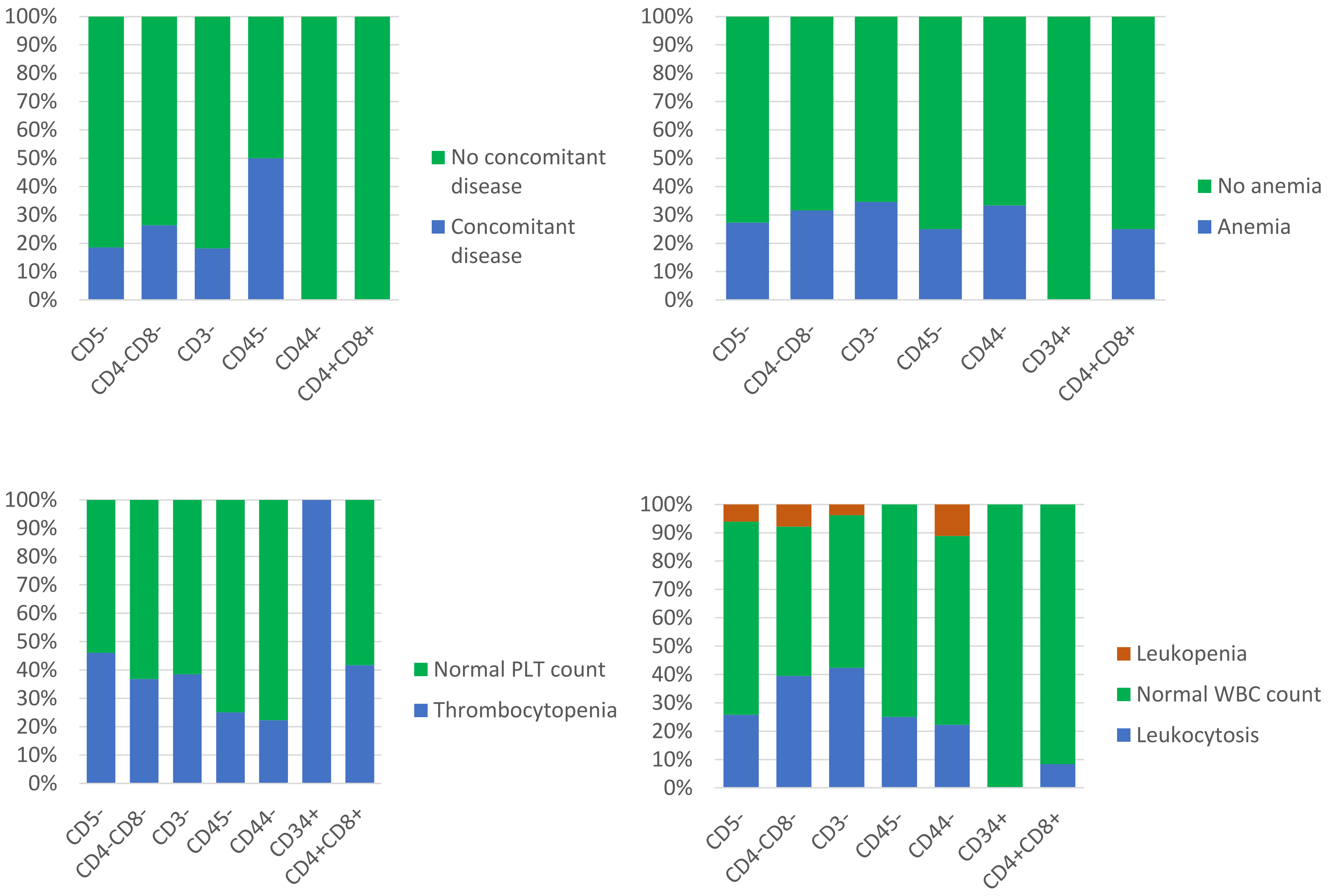
100%
90%
80%
70%
60%
50%
40%
30%
20%
10%
0%
CD5-
CD4-CD8-
CD3-
CD45-
CD44-
CD4+CD8+
No concomitant disease
Concomitant disease
CD34+
No anemia
Anemia
Normal PLT count
Thrombocytopenia
Leukopenia
Normal WBC count
Leukocytosis
