# Supplementary material for: Clinical and Clinical Pathological Presentation of 310 Dogs Affected by Lymphoma with Aberrant Antigen Expression Identified via Flow Cytometry
Source: Vet Sci. 2022 Apr 13;9(4):184. doi: 10.3390/vetsci9040184 (PMC9032799; doi:10.3390/vetsci9040184)
Supplement: Supplementary file 1 [file vetsci-09-00184-s001.zip › Supplementary Figure S2.pdf]

Supplementary Figure S2

Clinical and clinical-pathological data of 101 dogs diagnosed with T-zone lymphoma (TZL), according to specific phenotypic aberrancies

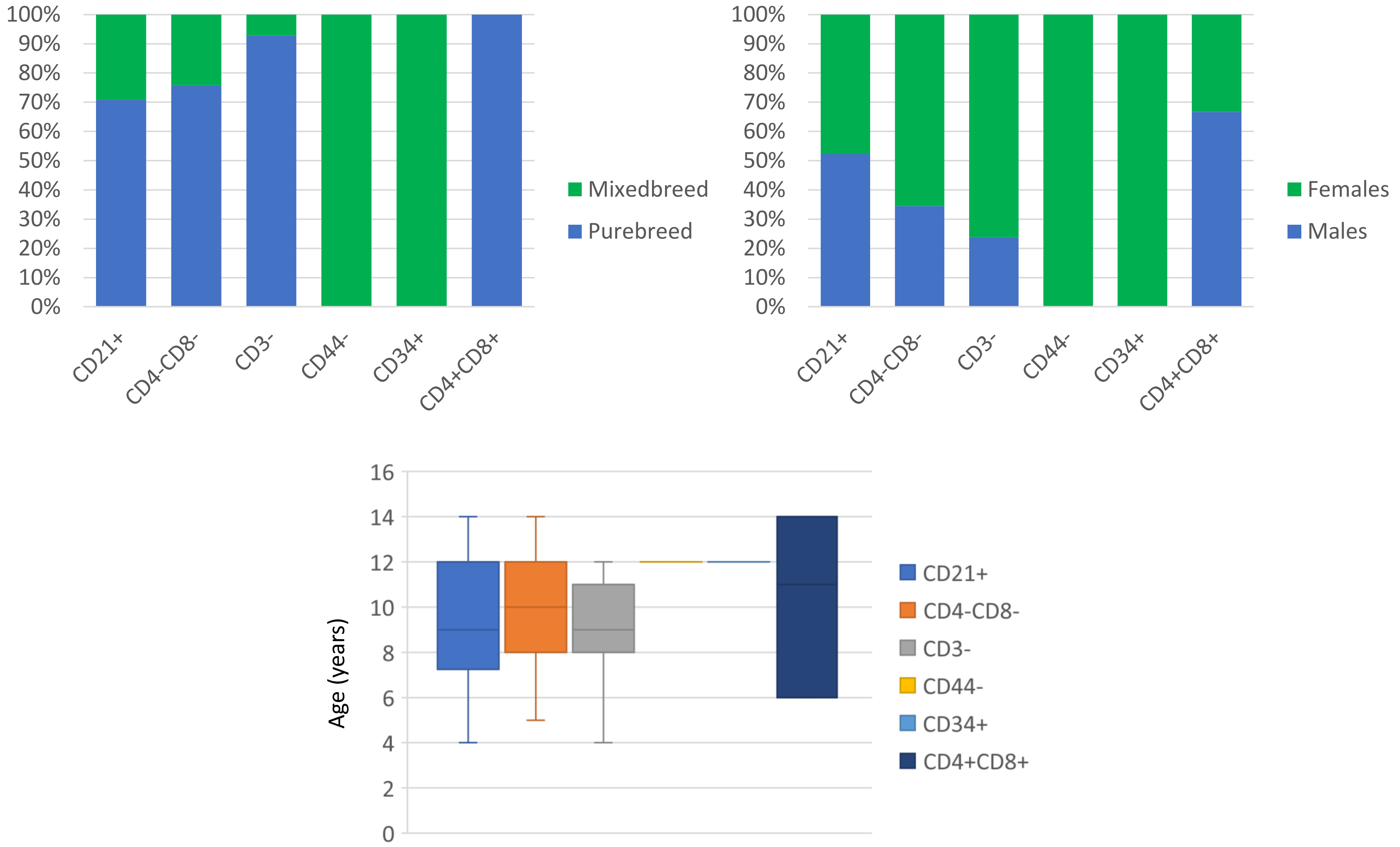

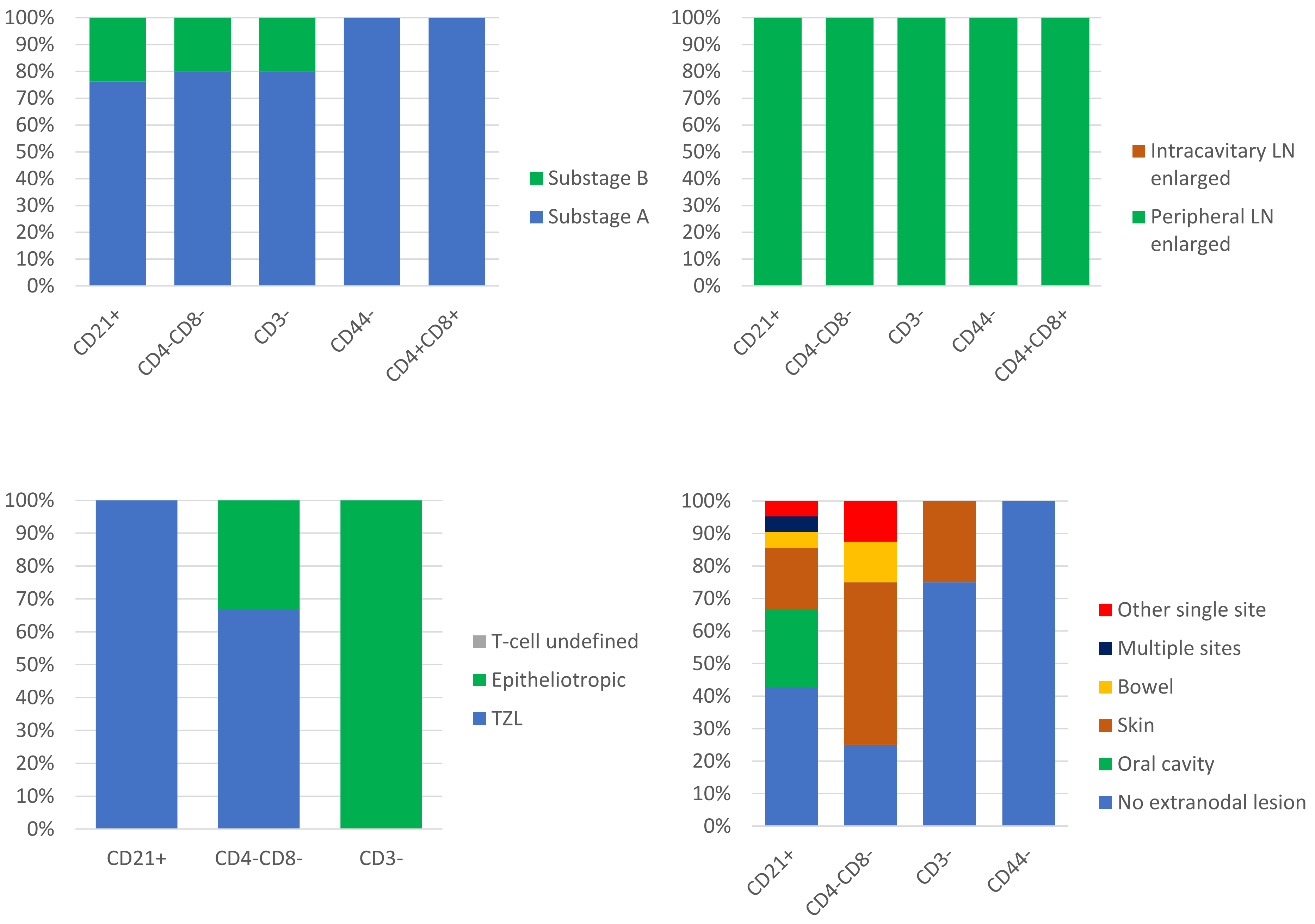
100%
90%
80%
70%
60%
50%
40%
30%
20%
10%
0%
CD21+
CD4-CD8-
CD3-
CD44-
CD4+CD8+
Substage B
Substage A
Intracavitary LN enlarged
Peripheral LN enlarged
T-cell undefined
Epitheliotropic
TZL
Other single site
Multiple sites
Bowel
Skin
Oral cavity
No extranodal lesion

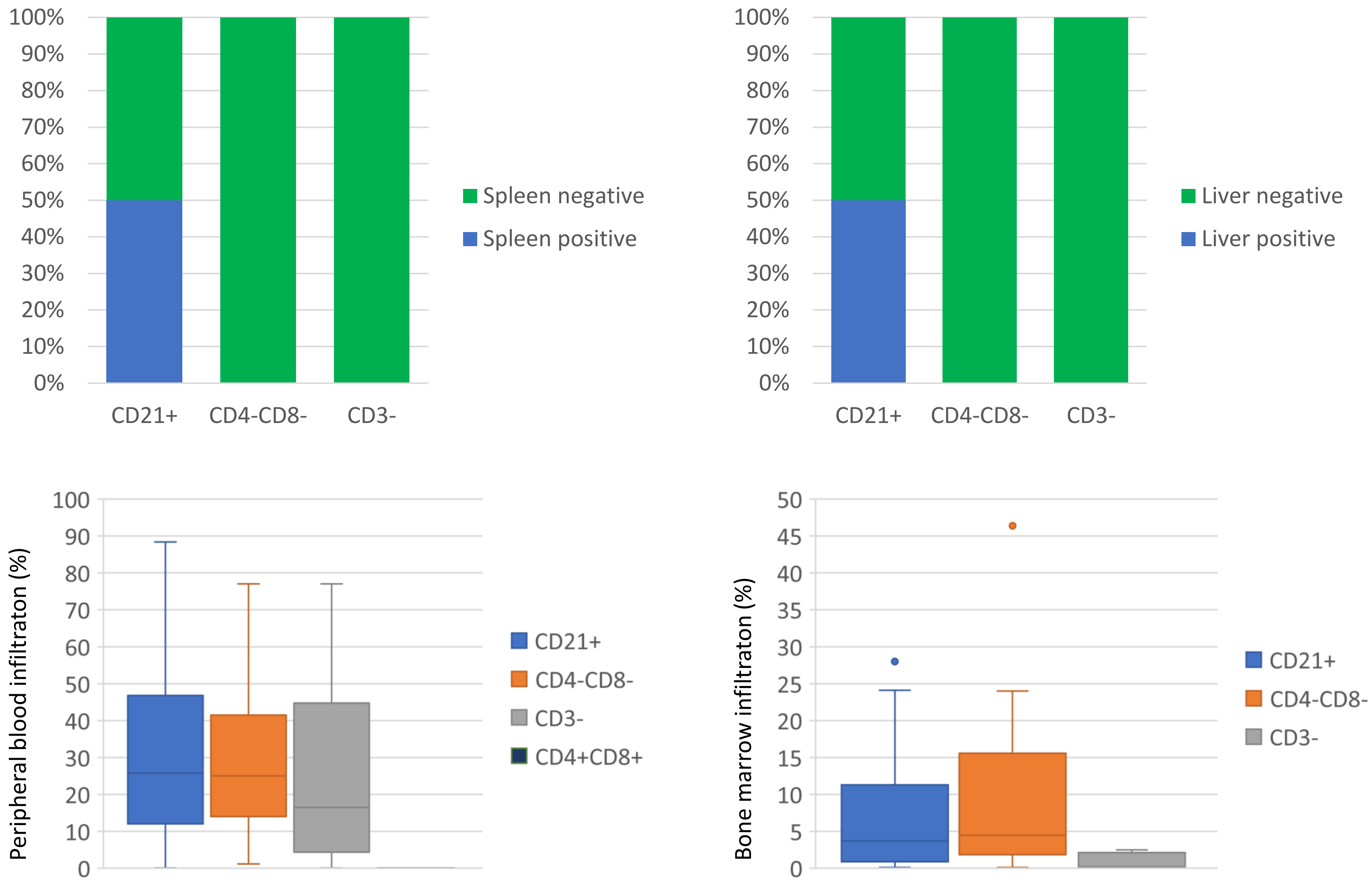
100%
90%
80%
70%
60%
50%
40%
30%
20%
10%
0%
CD21+
CD4-CD8-
CD3-
Spleen negative
Spleen positive
Liver negative
Liver positive
Peripheral blood infiltraton (%)
100
90
80
70
60
50
40
30
20
10
0
CD21+
CD4-CD8-
CD3-
CD4+CD8+
Bone marrow infiltraton (%)
50
45
40
35
30
25
20
15
10
5
0
CD21+
CD4-CD8-
CD3-

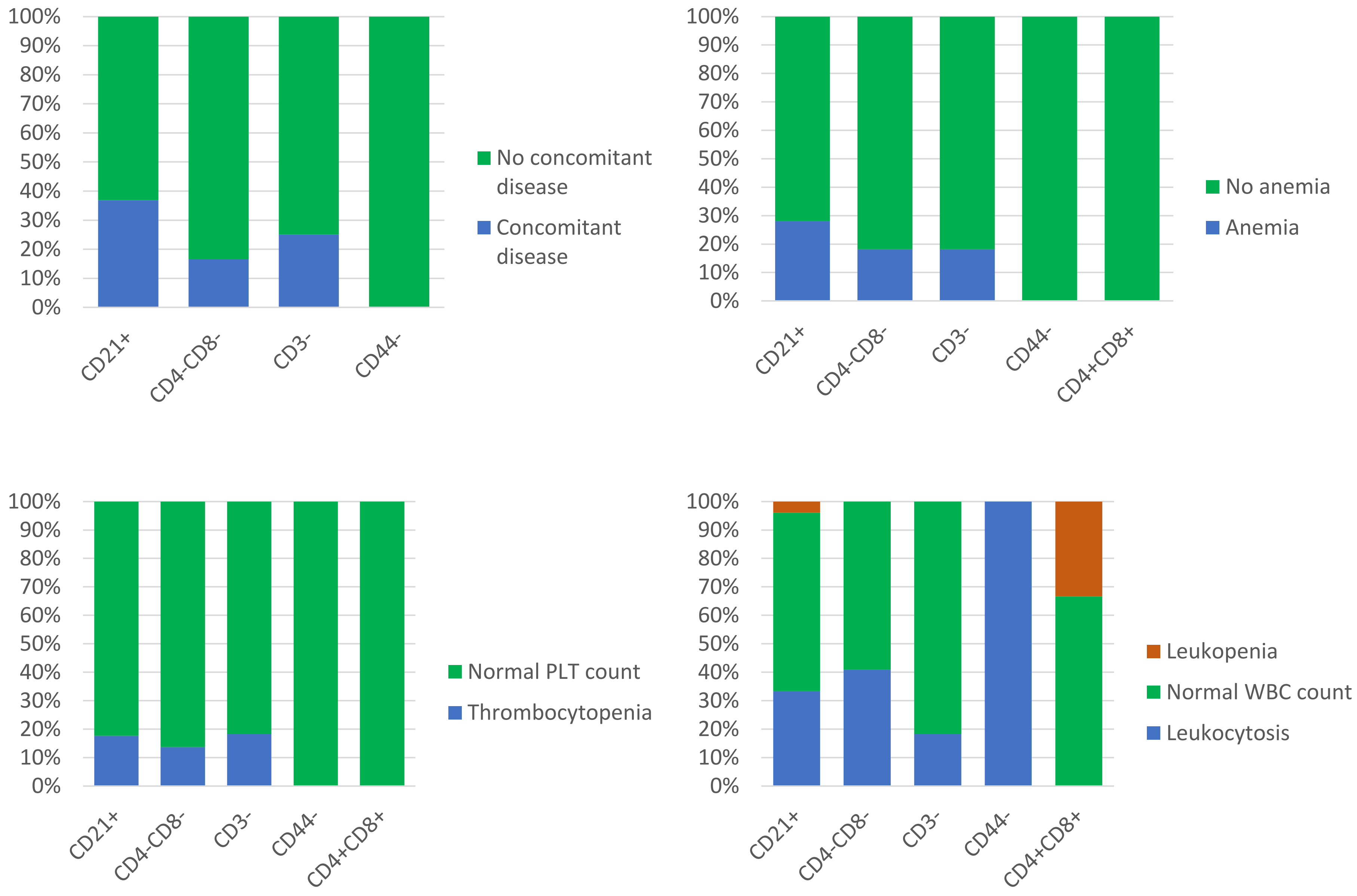
100%
90%
80%
70%
60%
50%
40%
30%
20%
10%
0%
CD21+
CD4-CD8-
CD3-
CD44-
CD4+CD8+
No concomitant disease
Concomitant disease
No anemia
Anemia
Normal PLT count
Thrombocytopenia
Leukopenia
Normal WBC count
Leukocytosis
