# Supplementary material for: Clinical and Clinical Pathological Presentation of 310 Dogs Affected by Lymphoma with Aberrant Antigen Expression Identified via Flow Cytometry
Source: Vet Sci. 2022 Apr 13;9(4):184. doi: 10.3390/vetsci9040184 (PMC9032799; doi:10.3390/vetsci9040184)
Supplement: Supplementary file 1 [file vetsci-09-00184-s001.zip › Supplementary Figure S3.pdf]

Supplementary Figure S3

Clinical and clinical-pathological data of 54 dogs diagnosed with B-cell lymphoma, according to specific phenotypic aberrancies

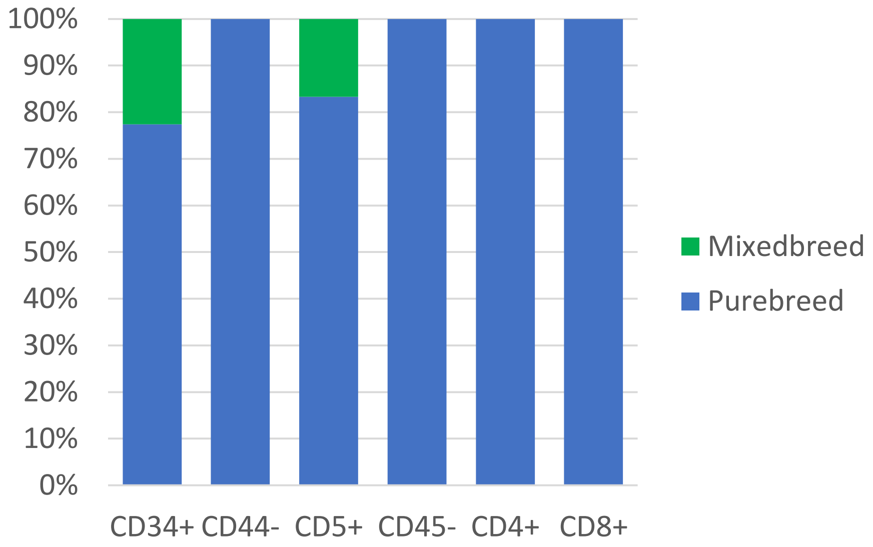

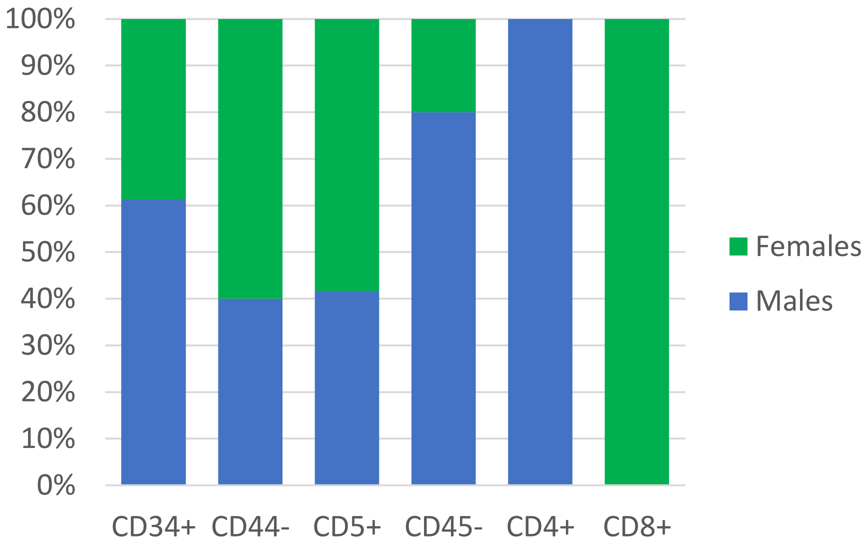

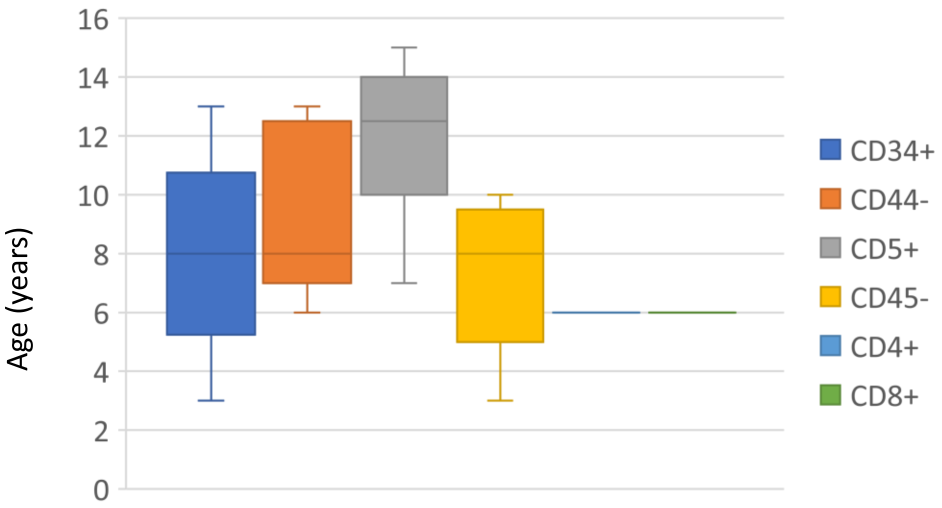

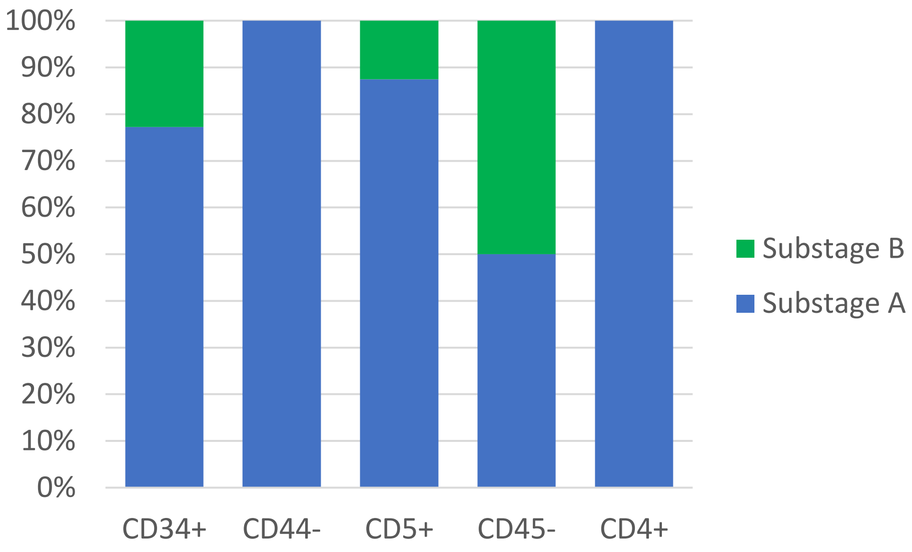
100%
90%
80%
70%
60%
50%
40%
30%
20%
10%
0%
CD34+
CD44-
CD5+
CD45-
CD4+
Substage B
Substage A

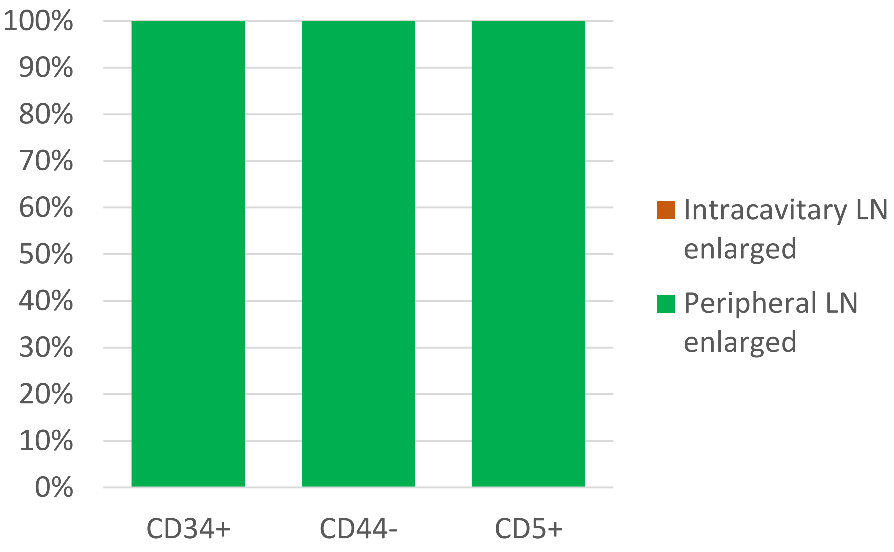
100%
90%
80%
70%
60%
50%
40%
30%
20%
10%
0%
CD34+
CD44-
CD5+
Intracavitary LN enlarged
Peripheral LN enlarged

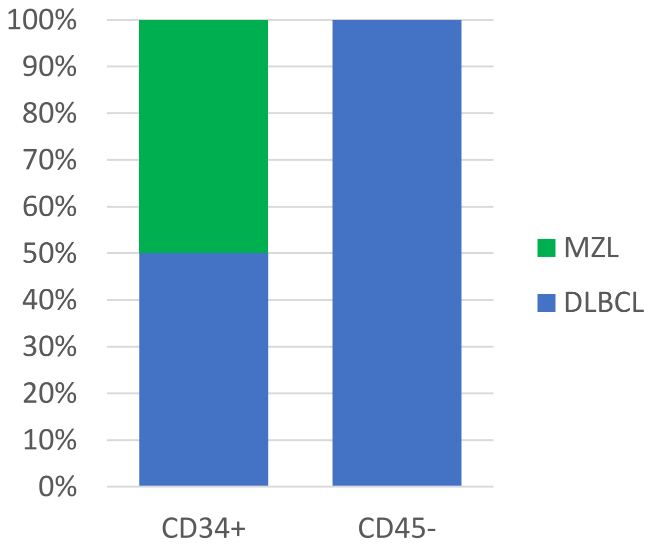
100%
90%
80%
70%
60%
50%
40%
30%
20%
10%
0%
CD34+
CD45-
MZL
DLBCL

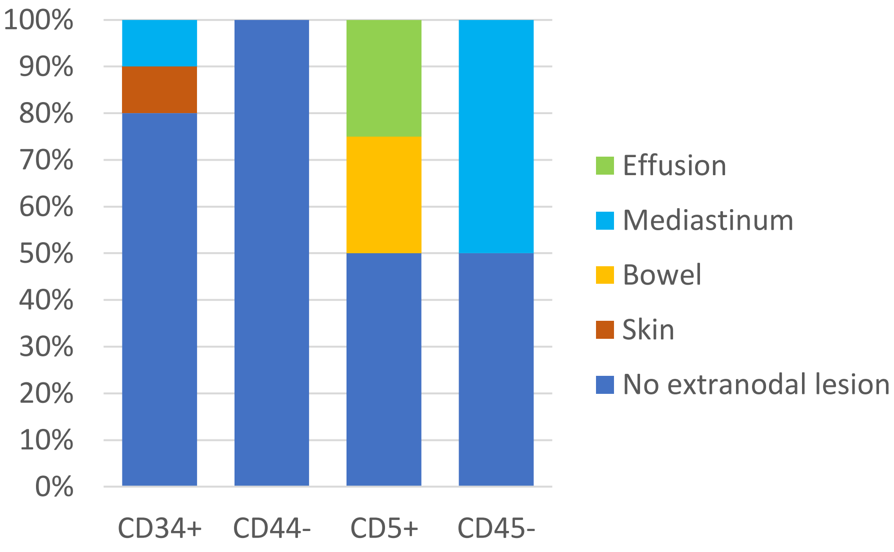
100%
90%
80%
70%
60%
50%
40%
30%
20%
10%
0%
CD34+
CD44-
CD5+
CD45-
Effusion
Mediastinum
Bowel
Skin
No extranodal lesion

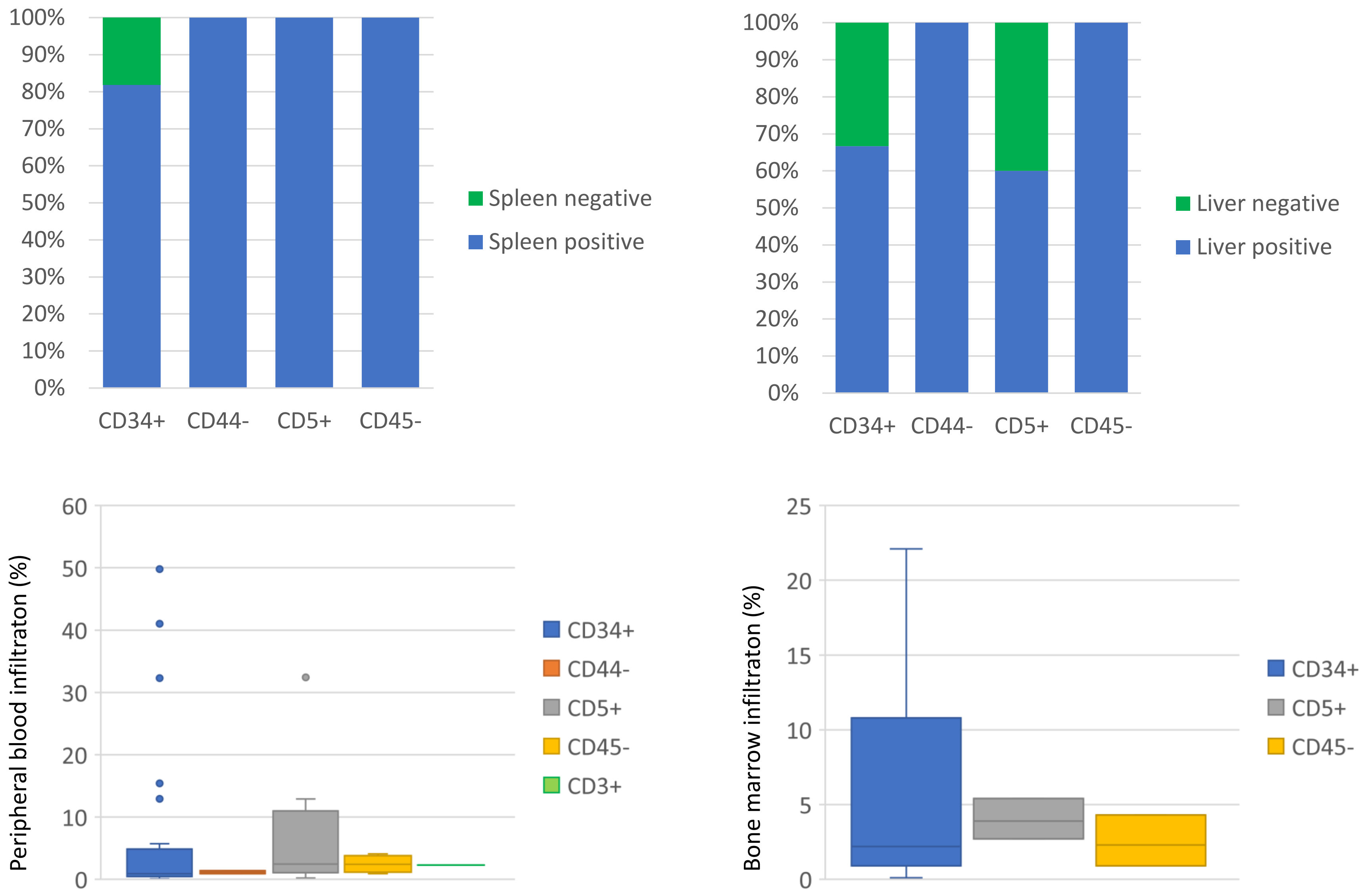
100%
90%
80%
70%
60%
50%
40%
30%
20%
10%
0%
CD34+
CD44-
CD5+
CD45-
Spleen negative
Spleen positive
Liver negative
Liver positive
Peripheral blood infiltraton (%)
60
50
40
30
20
10
0
CD34+
CD44-
CD5+
CD45-
CD3+
Bone marrow infiltraton (%)
25
20
15
10
5
0

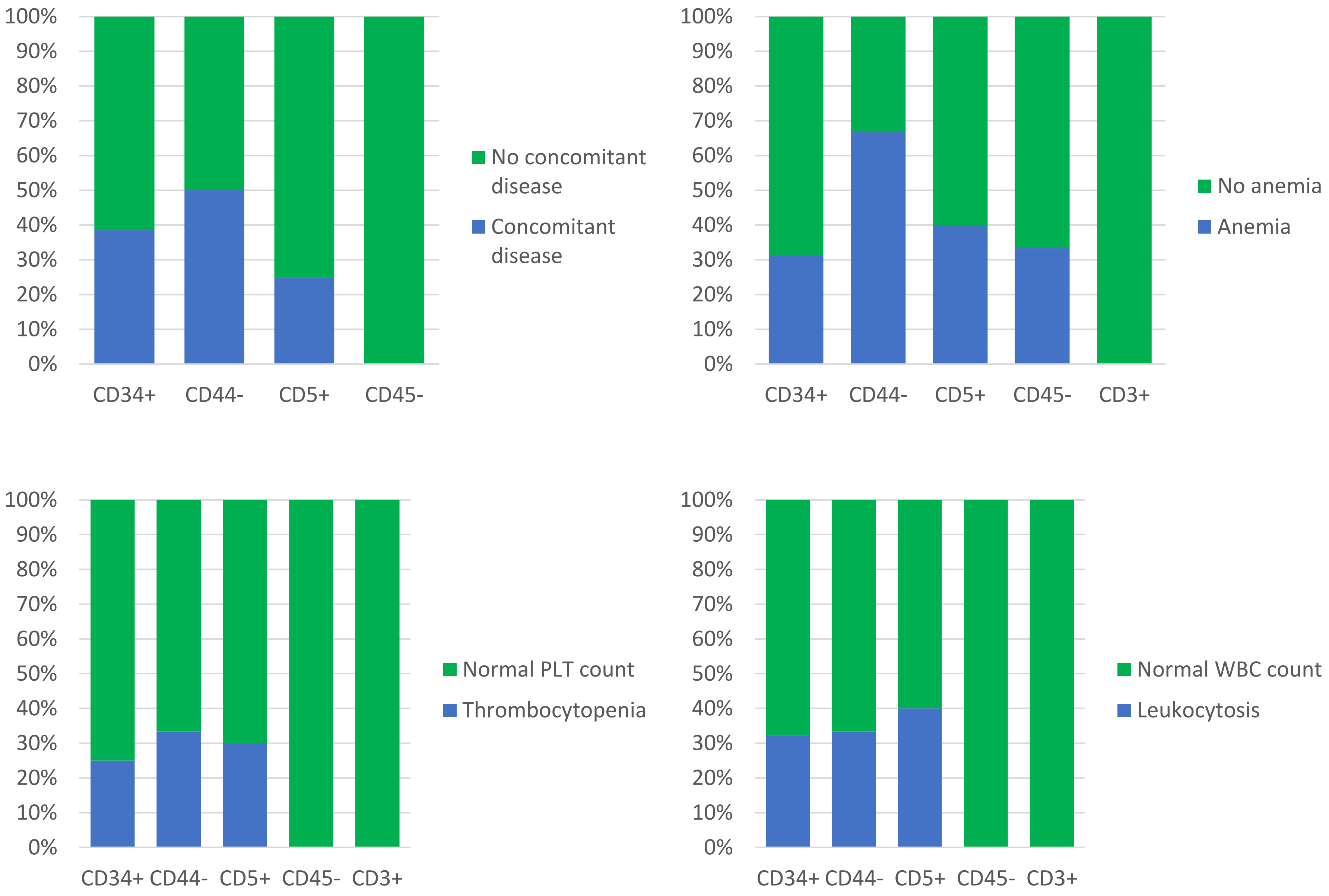

100%
90%
80%
70%
60%
50%
40%
30%
20%
10%
0%
CD34+
CD44-
CD5+
CD45-
No concomitant disease
Concomitant disease
CD3+
No anemia
Anemia
Normal PLT count
Thrombocytopenia
Normal WBC count
Leukocytosis
