# Supplementary material for: Clinical and Clinical Pathological Presentation of 310 Dogs Affected by Lymphoma with Aberrant Antigen Expression Identified via Flow Cytometry
Source: Vet Sci. 2022 Apr 13;9(4):184. doi: 10.3390/vetsci9040184 (PMC9032799; doi:10.3390/vetsci9040184)
Supplement: Supplementary file 1 [file vetsci-09-00184-s001.zip › Table S1.pdf]

**Table S1** number and frequencies of clinical and clinical-pathological features of 152 dogs with T-cell lymphoma not otherwise specified with aberrant antigen expression.

| | Number of aberrant cases out of total cases tested for the antigen | | | | | | | |
|---|---|---|---|---|---|---|---|---|
| | CD5- | CD4-CD8- | CD3- | CD45- | CD44- | CD34+ | CD4+CD8+ | CD21+ |
| Pure breed | 64/108 | 39/112 | 23/110 | 7/116 | 10/53 | 1/96 | 13/112 | 1/115 |
| Mixed breed | 17/28 | 11/28 | 8/28 | 1/28 | 2/12 | 0/24 | 3/28 | 0/27 |
| Males | 48/76 | 25/76 | 16/74 | 4/78 | 9/34 | 1/67 | 9/76 | 1/77 |
| Females | 33/61 | 25/65 | 16/64 | 4/67 | 3/30 | 0/53 | 7/65 | 0/66 |
| Substage a | 46/69 | 21/71 | 11/66 | 2/71 | 4/33 | 1/58 | 7/71 | 1/71 |
| Substage b | 16/36 | 17/34 | 11/36 | 3/38 | 4/19 | 0/32 | 5/34 | 0/37 |
| No LN enlargement | 4/4 | 1/3 | 0/1 | 0/4 | 1/2 | 0/1 | 0/3 | 0/4 |
| Peripheral LNs | 58/89 | 26/90 | 17/90 | 4/92 | 6/47 | 1/81 | 10/90 | 1/92 |
| Intracavitary LNs | 5/17 | 10/17 | 6/17 | 1/18 | 2/7 | 0/15 | 3/17 | 0/18 |
| PTCL | 7/9 | 6/8 | 2/9 | 0/9 | 0/4 | 0/7 | 1/8 | 0/9 |
| Mycosis fungoides | 2/2 | 0/2 | 1/1 | 0/2 | 0/1 | - | 0/2 | 0/2 |
| Small lymphocytic | 0/2 | 2/2 | 0/2 | 0/2 | 0/1 | 0/2 | 0/2 | 0/2 |
| Non-epitheliotropic | 1/1 | 0/1 | - | 0/1 | - | - | 0/1 | 0/1 |
| Lymphoblastic | 0/1 | 0/1 | 0/1 | 0/1 | 1/1 | 0/1 | 1/1 | 0/1 |
| Spleen positive | 9/14 | 5/15 | 2/14 | 0/15 | 1/5 | 0/13 | 2/15 | 0/14 |
| Spleen negative | 17/27 | 10/25 | 5/23 | 0/27 | 1/10 | 0/20 | 2/25 | 0/27 |
| Liver positive | 7/13 | 6/14 | 3/13 | 0/14 | 1/7 | 0/13 | 2/14 | 0/14 |
| Liver negative | 17/27 | 9/25 | 5/23 | 0/27 | 1/9 | 0/20 | 2/25 | 0/27 |
| No extranodal site | 12/18 | 3/19 | 5/18 | 1/19 | 0/7 | 0/18 | 1/19 | 0/19 |
| Oral cavity | 1/5 | 3/6 | 1/6 | 0/6 | 0/1 | 0/4 | 2/6 | 0/6 |
| Skin | 9/13 | 8/13 | 3/11 | 0/13 | 0/3 | 0/7 | 0/13 | 0/13 |
| Bowel | 2/3 | 1/2 | 0/2 | 0/3 | 1/2 | 0/2 | 0/2 | 0/3 |
| Mediastinum | 8/12 | 5/13 | 0/11 | 0/13 | 1/6 | 0/10 | 2/13 | 0/13 |
| Effusion | 3/12 | 7/13 | 6/13 | 1/13 | 3/4 | 0/13 | 4/13 | 0/12 |
| Multiple sites | 3/5 | 1/3 | 2/4 | 0/5 | 1/2 | 0/3 | 2/3 | 0/5 |
| Other single site | 1/1 | 0/1 | 0/1 | 0/1 | - | 0/1 | 0/1 | 0/1 |
| Concomitant disease | 5/8 | 5/10 | 2/10 | 1/10 | 0/6 | 0/7 | 0/10 | 0/10 |
| No concomitant disease | 22/37 | 14/37 | 9/35 | 1/40 | 2/13 | 0/30 | 6/37 | 0/40 |
| Anemia | 18/33 | 12/32 | 9/33 | 1/35 | 3/16 | 0/28 | 3/32 | 0/35 |
| No anemia | 48/78 | 26/81 | 17/78 | 3/82 | 6/37 | 1/69 | 9/81 | 0/80 |
| Thrombocytopenia | 29/46 | 14/46 | 10/44 | 1/47 | 2/18 | 1/40 | 5/46 | 0/46 |
| Normal PLT count | 34/62 | 24/64 | 16/64 | 3/67 | 7/33 | 0/55 | 7/64 | 0/66 |
| Leukocytosis | 17/31 | 15/33 | 11/32 | 1/33 | 2/12 | 0/28 | 1/33 | 0/32 |
| Normal WBC count | 45/76 | 20/74 | 14/73 | 3/78 | 6/38 | 1/64 | 11/74 | 0/77 |
| Leukopenia | 4/4 | 3/6 | 1/6 | 0/6 | 1/3 | 0/5 | 0/6 | 0/6 |

LN=lymph node. PTCL=Peripheral T-Cell Lymphoma
