# Supplementary material for: Clinical and Clinical Pathological Presentation of 310 Dogs Affected by Lymphoma with Aberrant Antigen Expression Identified via Flow Cytometry
Source: Vet Sci. 2022 Apr 13;9(4):184. doi: 10.3390/vetsci9040184 (PMC9032799; doi:10.3390/vetsci9040184)
Supplement: Supplementary file 1 [file vetsci-09-00184-s001.zip › Table S2.pdf]

**Table S2** number and frequencies of clinical and clinical-pathological features of 101 dogs with T-zone lymphoma with aberrant antigen expression.

| | Number of aberrant cases out of total cases tested for the antigen | | | | | | |
|---|---|---|---|---|---|---|---|
| | CD21+ | CD4-CD8- | CD3- | CD44- | CD34+ | CD5- | CD4+CD8+ |
| Pure breed | 46/70 | 22/70 | 13/63 | 0/52 | 0/71 | 0/73 | 3/70 |
| Mixed breed | 19/24 | 7/21 | 1/19 | 2/19 | 1/23 | 0/23 | 0/21 |
| Males | 34/48 | 10/45 | 9/41 | 0/37 | 0/48 | 0/48 | 2/45 |
| Females | 31/46 | 19/46 | 5/41 | 2/33 | 1/46 | 0/48 | 1/46 |
| Substage a | 32/51 | 16/50 | 8/44 | 1/37 | 0/51 | 0/53 | 1/50 |
| Substage b | 10/12 | 4/12 | 2/11 | 0/10 | 0/12 | 0/12 | 0/12 |
| Peripheral LNs | 49/69 | 19/67 | 10/58 | 1/51 | 0/69 | 0/72 | 3/67 |
| Intracavitary LNs | 0/1 | - | 0/1 | 0/1 | - | - | - |
| TZL | 9/13 | 2/13 | 0/10 | 0/10 | 0/14 | 0/15 | 0/13 |
| Epitheliotropic | 0/1 | 1/1 | 1/1 | - | - | 0/1 | 0/1 |
| T-cell, undefined | 0/1 | 0/1 | 0/1 | 0/1 | - | 0/1 | 0/1 |
| Spleen positive | 7/12 | 0/10 | 0/9 | 0/8 | 0/11 | 0/12 | 0/10 |
| Spleen negative | 7/10 | 3/11 | 3/8 | 0/5 | 0/10 | 0/11 | 0/11 |
| Liver positive | 7/11 | 0/9 | 0/7 | 0/7 | 0/10 | 0/10 | 0/9 |
| Liver negative | 7/11 | 3/12 | 3/11 | 0/6 | 0/11 | 0/13 | 0/12 |
| No extranodal site | 9/15 | 2/15 | 3/13 | 1/13 | 0/16 | 0/17 | 0/15 |
| Oral cavity | 5/6 | 0/5 | 0/5 | 0/4 | 0/6 | 0/6 | 0/5 |
| Skin | 4/7 | 4/7 | 1/7 | 0/4 | 0/6 | 0/7 | 0/7 |
| Bowel | 1/1 | 1/1 | 0/1 | - | 0/1 | 0/1 | 0/1 |
| Multiple sites | 1/1 | 0/2 | 0/2 | 0/2 | 0/2 | 0/2 | 0/2 |
| Other single site | 1/1 | 1/1 | 0/1 | 0/1 | 0/1 | 0/1 | 0/1 |
| Concomitant disease | 7/10 | 1/12 | 1/10 | 0/8 | 0/12 | 0/12 | 0/12 |
| No concomitant disease | 12/18 | 5/17 | 3/16 | 1/13 | 0/16 | 0/18 | 0/17 |
| Anemia | 14/20 | 4/18 | 2/14 | 0/13 | 0/20 | 0/20 | 0/18 |
| No anemia | 36/55 | 18/54 | 9/49 | 1/39 | 0/64 | 0/56 | 1/54 |
| Thrombocytopenia | 9/10 | 3/10 | 2/8 | 0/6 | 0/10 | 0/10 | 0/10 |
| Normal PLT count | 42/66 | 19/63 | 9/56 | 1/47 | 0/65 | 0/67 | 1/63 |
| Leukocytosis | 17/27 | 9/25 | 2/21 | 1/22 | 0/26 | 0/26 | 0/25 |
| Normal WBC count | 32/47 | 13/47 | 9/42 | 0/31 | 0/47 | 0/49 | 2/47 |
| Leukopenia | 2/2 | 0/1 | 0/1 | - | 0/2 | 0/2 | 0/1 |

LN=lymph node. TZL=T-zone Lymphoma
