# Supplementary material for: Clinical and Clinical Pathological Presentation of 310 Dogs Affected by Lymphoma with Aberrant Antigen Expression Identified via Flow Cytometry
Source: Vet Sci. 2022 Apr 13;9(4):184. doi: 10.3390/vetsci9040184 (PMC9032799; doi:10.3390/vetsci9040184)
Supplement: Supplementary file 1 [file vetsci-09-00184-s001.zip › Table S3.pdf]

Table S3 number and frequencies of clinical and clinical-pathological features of 54 dogs with B-cell lymphoma with aberrant antigen expression.

	Number of aberrant cases out of total cases tested for the antigen						
	CD34+	CD44-	CD5+	CD45-	CD3+	CD4+	CD8+
Pure breed	24/40	5/15	10/40	4/41	0/24	1/30	1/30
Mixed breed	7/9	0/3	2/9	0/9	0/5	0/5	0/5
Males	19/28	2/10	5/28	4/29	0/17	1/21	0/21
Females	12/22	3/9	7/22	1/22	0/13	0/15	1/15
Substage a	17/27	3/12	7/27	1/27	0/16	1/20	0/20
Substage b	5/6	0/3	1/6	1/6	0/3	0/5	0/5
DLBCL	2/3	0/2	0/3	1/3	0/2	0/2	0/2
MZL	2/3	0/2	0/2	0/3	0/2	0/3	0/3
Spleen positive	9/15	1/5	5/15	1/15	0/6	0/10	0/10
Spleen negative	2/2	0/1	0/2	0/2	0/1	0/2	0/2
Liver positive	6/10	1/3	3/10	1/10	0/4	0/6	0/6
Liver negative	3/5	0/2	2/5	0/5	0/2	0/5	0/5
No extranodal site	8/11	1/7	2/11	1/11	0/6	0/9	0/9
Skin	1/1	-	0/1	0/1	-	0/1	0/1
Bowel	0/1	-	1/1	0/1	0/1	0/1	0/1
Mediastinal	1/1	-	0/1	1/1	0/1	0/1	0/1
Effusion	0/1	0/1	1/1	0/1	0/1	0/1	0/1
Concomitant disease	5/7	1/3	1/7	0/7	0/4	0/5	0/5
No concomitant disease	8/12	1/6	3/12	1/12	0/7	0/9	0/9
Anemia	9/12	2/5	4/12	1/12	0/8	0/8	0/8
No anemia	20/29	1/10	6/29	2/30	1/17	0/21	0/21
Thrombocytopenia	7/11	1/2	3/11	0/11	0/5	0/5	0/5
Normal PLT count	21/29	2/12	7/29	3/30	1/19	0/23	0/23
Leukocytosis	9/12	1/3	4/12	0/12	0/6	0/7	0/7
Normal WBC count	19/28	2/11	6/28	3/29	1/18	0/21	0/21

LN=lymph node. DLBCL=Diffuse Large B-Cell Lymphoma. MZL=Marginal Zone Lymphoma
