# Supplementary material for: Clinical and Clinical Pathological Presentation of 310 Dogs Affected by Lymphoma with Aberrant Antigen Expression Identified via Flow Cytometry
Source: Vet Sci. 2022 Apr 13;9(4):184. doi: 10.3390/vetsci9040184 (PMC9032799; doi:10.3390/vetsci9040184)
Supplement: Supplementary file 1 [file vetsci-09-00184-s001.zip › Table S4.pdf]

Table S4 age, blood and marrow infiltration in 310 dogs with lymphoma with aberrant antigen expression, according to lymphoma subtype.

Aberrancy		Median; min-max		
		Age (years)	PB infiltration (%)	BM infiltration (%)
			**T-NOS lymphoma**	
Loss of CD5				
	Present	7; 3-13	2.8; 0.1-60.0	1.2; 0.1-72.5
	Absent	8; 1-16	2.9; 0.1-68.0	1.2; 0.1-80.1
CD4/CD8 double negative				
	Present	8; 1-14	1.4; 0.1-60.0	0.9; 0.1-44.8
	Absent	8; 1-16	3.2; 0.1-68.0	1.5; 0.1-80.1
Loss of CD3				
	Present	8; 1-16	2.9; 0.1-40.0	1.0; 0.1-80.1
	Absent	8; 1-14	2.9; 0.1-68.0	1.3; 0.1-72.5
Loss of CD45				
	Present	9; 5-12	13.6; 0.1-40.3	1.2; 0.1-44.8
	Absent	8; 1-16	2.7; 0.1-68.0	1.1; 0.1-80.1
Loss of CD44				
	Present	8.5; 1-16	3.4; 0.1-24.2	1.2; 0.9-20.0
	Absent	8; 1-14	5.8; 0.1-60.0	5.3; 0.1-80.1
Expression of CD34				
	Present	4	1.9	0.9
	Absent	8; 1-14	2.9; 0.1-68.0	1.9; 0.1-80.1
CD4/CD8 double positive				
	Present	9; 1-16	2.9; 0.1-68.0	1.1; 0.1-3.3
	Absent	8; 1-14	2.7; 0.1-60.0	1.2; 0.1-80.1
Expression of CD21				
	Present	8	-	-
	Absent	8; 1-16	2.9; 0.1-68.0	1.1; 0.1-80.1
			**T-zone lymphoma**	
Expression of CD21				
	Present	9; 4-14	25.8; 0.1-88.4	3.7; 0.1-28.0
	Absent	11; 5-15	17.0; 0.1-77.0	2.0; 0.1-46.4
CD4/CD8 double negative				
	Present	10; 5-14	25.0; 1.2-77.0	4.5; 0.1-46.4
	Absent	10; 4-14	23.9; 0.1-88.4	2.0; 0.1-28.0
Loss of CD3				
	Present	9; 4-12	16.5; 0.1-77.0	0.2; 0.1-2.5
	Absent	10; 5-14	28.3; 2.0-77.0	4.8; 0.1-46.4
Loss of CD44				
	Present	12, 12	-	-
	Absent	9; 5-14	31.8; 0.9-88.4	2.8; 0.1-46.4
Expression of CD34				
	Present	12	-	-
	Absent	10; 4-15	22.8; 0.1-88.4	2.6; 0.1-46.4
Loss of CD5				
	Present	-	-	-
	Absent	10; 4-15	22.0; 0.1-88.4	2.7; 0.1-46.4
CD4/CD8 double positive				
	Present	11; 6-14	0.1	-
	Absent	10; 4-14	25.4; 0.1-88.4	2.5; 0.1-46.4
			**B-cell lymphoma**	
Expression of CD34				
	Present	8; 3-13	0.9; 0.1-49.8	2.2; 0.1-22.1
	Absent	10; 3-15	1.9; 0.2-32.4	3.9; 2.3-5.4
Loss of CD44				
	Present	8; 6-13	1.2; 0.9-1.4	-
	Absent	8; 3-12	0.9; 0.1-15.4	2.5; 0.1-15.5
Expression of CD5				
	Present	12.5; 7-15	2.5; 0.2-32.4	3.9; 2.7-5.4
	Absent	8; 3-13	0.9; 0.1-49.8	2.9; 0.1-22.1
Loss of CD45				
	Present	8; 3-10	2.4; 0.9-4.1	2.3; 0.9-4.3

	Absent	8; 3-15	1.2; 0.1-49.8	3.5; 0.1-22.1
Expression of CD3				
	Present	-	2.3	-
	Absent	8; 3-14	1.4; 0.1-15.4	3.4; 0.1-15.5
Expression of CD4				
	Present	6	-	-
	Absent	8; 3-15	1.4; 0.1-41.0	3.7; 0.1-22.1
Expression of CD8				
	Present	6	-	-
	Absent	8; 3-15	1.4; 0.1-41.0	3.7; 0.1-22.1

T-NOS= T-cell lymphoma not otherwise specified. PB=peripheral blood. BM=bone marrow.
